# Computationally derived RNA polymerase III promoters enable maize genome editing

**DOI:** 10.3389/fpls.2025.1540425

**Published:** 2025-03-19

**Authors:** Ervin D. Nagy, Ian W. Davis, Shanshan Song, Valerie No, Chenxi Wu, Lisa Kanizay, Sarah Turner-Hissong, Hong Li, Xudong Ye, Jeffrey C. Berry, Brandi Chiapelli, Jennifer P. C. To, Matthew S. Marengo

**Affiliations:** Plant Biotechnology, Bayer Crop Science, Chesterfield, MO, United States

**Keywords:** RNA polymerase III, promoter, CRISPR, LbCas12a, maize (*Zea mays* L.)

## Abstract

CRISPR endonucleases require cognate non-coding RNA species for site-specific activity. These RNA species are typically expressed using endogenous RNA polymerase III (Pol III) promoters compatible with the host species. This study describes applications of novel Pol III promoters, which were computationally derived from a training set of monocot U6 and U3 promoters. These promoters enabled genome editing in maize protoplast cells and maize plants. Out of 37 novel promoters, 27 performed similarly to a control U6 promoter. Multiplexing five novel promoters in one construct enabled simultaneous editing of the maize genome at 27 unique sites in a single plant. Moreover, repeating the same CRISPR RNA (crRNA) with multiple novel promoters improved editing up to three-fold at a low-efficiency target site in maize plants. The ability to computationally derive novel Pol III promoters on-demand increases genome editing flexibility and efficiency in maize.

## Introduction

In recent decades, genome editing has revolutionized crop biotechnology and breeding. Clustered Regularly Interspaced Short Palindromic Repeats (CRISPR) systems, including CRISPR/Cas9 (Jinek et al., 2012) and CRISPR/Cas12a (Zetsche et al., 2015), have gained prominence due to their efficacy and versatility over alternative editing technologies. The site-specific endonuclease activity of CRISPR systems requires the formation of a complex with associated non-coding RNA species. The CRISPR/Cas9 system forms a complex with two RNA species: a CRISPR RNA (crRNA) and a trans-acting CRISPR RNA (tracrRNA). The artificial chimeric derivative of these species is called a single-guide RNA (sgRNA). In contrast, the CRISPR/Cas12a system requires only a crRNA for activity. These crRNA species include a conserved repeat, which is required for association with the Cas12a enzyme, followed by a variable spacer that corresponds to the editing target site (Zetsche et al., 2015).

In order to use these CRISPR systems to edit eukaryotic genomes, the heterologous endonuclease gene is transcribed by RNA polymerase II (Pol II), translated to protein in the cytoplasm, and targeted to the nucleus by nuclear localization signals (NLSs). In contrast, the CRISPR RNA species are typically retained in the nucleus after transcription, where they assemble with the endonuclease and act upon the genomic DNA. While most RNA species are transported out of the nucleus for activity (such as mRNAs, tRNAs and rRNAs), small nuclear RNAs (snRNAs) contributing to spliceosome ribonucleo-protein (RNP) complexes are retained therein. Some snRNAs, such as U6 and (in plants) U3 snRNAs, are transcribed by RNA polymerase III (Pol III) (Waibel and Filipowicz, 1990; Marshallsay et al., 1992). Pol III promoters are shorter and less complex than Pol II promoters, and the resulting transcripts are not subject to 7-methylguanosine (m7G) capping or polyadenylation (Gao et al., 2018). These features make Pol III promoters useful tools for heterologous expression of non-coding nuclear RNA species (Ma et al., 2014). While it’s important to note that CRISPR RNA species can also be expressed by alternative means, such as Pol II or viral promoters (Gao and Zhao, 2014; Tang et al., 2016; Xu et al., 2019; Ellison et al., 2020; Li et al., 2021), Pol III promoters have remained a standard tool for CRISPR systems (Kor et al., 2023).

Eukaryotic U3 and U6 promoters have two major conserved regions, the -30 bp TATA box and the upstream sequence element (USE, consensus TCCCACATCG), which are required and sufficient for transcription in dicotyledonous plant species. The spacing between these two elements is a major determinant for recognition by Pol III (Waibel and Filipowicz, 1990; Marshallsay et al., 1992). Expression in monocotyledonous species requires additional monocot-specific promoter element(s) 5’ of the USE (MSPs, consensus RGCCCR). The strength of these MSP elements is a major determinant of the overall activity for these promoters (Connelly et al., 1994).

While snRNA promoters can drive transcription across different taxa, their efficacies can be suboptimal when used in distantly related species (Connelly et al., 1994; Liu et al., 2020; Massel et al., 2022). The endogenous maize U6 (Svitashev et al., 2015; Zhu et al., 2016; Kouranov et al., 2022) and U3 (Liang et al., 2014) promoters have been extensively used for maize genome editing in the past decade. While the efficacies of these promoters are sufficient for many purposes, complex editing will require more than these two promoters. For example, additional novel promoters could better facilitate highly multiplexed genome editing, in which entire physiological pathways or large gene families are targeted simultaneously (Zhou et al., 2023). Specifically, repeated elements in expression vectors can increase clonal instability and risk of silencing in planta (Sinden et al., 1999; Assaad et al., 1993). Diversification of Pol III promoters, by minimizing redundancy of expression elements, can mitigate these issues effectively. Computational derivation of such promoters on demand can be an effective approach to increase diversity of promoters.

The goal of this study was to expand and diversify the pool of Pol III promoters for maize genome editing. A computational algorithm was previously used to derive new expression elements, including promoters and introns, for Pol II genes in crops (To et al., 2021). Using the same method, we derived thirty-seven novel Pol III promoters for maize expression and tested their efficacies for both simplex and multiplex genome editing.

## Materials and methods

### Mining endogenous U6 promoters

**Figure 1:**
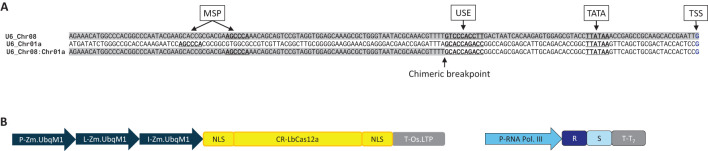
**(A)** Structures of the native U6 promoters Chr08 and Chr01 of maize and their chimeric derivative, Chr08:Chr01, used as a positive control throughout this study. 160 bp of the promoter region immediately upstream of the transcription start site (TSS, blue) included three conserved elements (underlined) previously shown to be important for expression: the TATA box, the upstream sequence element (USE), and the monocot-specific promoter element (MSP). **(B)** Expression cassettes for LbCas12a and its cognate crRNA for testing endogenous and computationally derived Pol III promoters for genome editing. P, promoter; L, leader (5’ untranslated region); I, intron; NLS, nuclear localization signal; CR, coding region; T, terminator (3’ untranslated region); R, crRNA repeat; S, crRNA spacer.

Altogether 42 U6 promoters and 24 U3 promoters from maize and six other monocotyledonous species were mined from public and Bayer proprietary databases as a training set for a proprietary generative machine learning model. An arbitrarily selected subset of endogenous U6 promoters were directly tested (**Supplementary Material S1**). Three endogenous maize U6 promoters, one each from chromosomes 1, 2 and 3 (referenced below as Chr01, Chr02 and Chr03, respectively), were tested in different length variants (160 bp and 400 bp) for genome editing. Based on inspection for TATA, USE, and MSP elements, 160 bp was chosen for minimal length variants for these promoters. The 400 bp variants were included to test for other potential upstream element(s) that could contribute to the promoter activity. A chimeric derivative between Chr08 and Chr01 U6 promoters (160 bp) was used as a positive control (**Figure 1A**) across several experiments in this investigation.

### Computational sequence design

Novel Pol III promoters were computationally derived as previously described for Pol II promoters (To et al., 2021). Briefly, from the training set promoters, we extracted genomic sequence from -500 to +50 bases relative to the transcription start site (TSS). We identified statistically overrepresented sequence motifs that are likely to contribute to promoter function using the POWRS v1.2 algorithm, using “—bins 5” and otherwise default settings (Davis et al., 2012). As expected, the primary motifs were the TATA box, the MSP, and the USE. Multiple sequence alignment using MUSCLE v3.8 (Edgar, 2004) confirmed that lengths of intervening sequences between USE and TATA and between TATA and TSS were extremely consistent. The endogenous monocot promoters and the putative sequence motifs were used to train a proprietary generative machine learning model. The sequence design algorithm has been described in detail (Davis and Elich, 2025). In brief, the algorithm uses a scoring function to estimate how promoter-like a sequence is. The function has terms for position-specific 6-mer frequency in the training set, position-specific 6-mer enrichment relative to non-training-set promoters, and dinucleotide entropy. Starting from a random sequence, we randomly placed overrepresented motifs, then iteratively improved the sequence according to the scoring function, via random mutation with simulated annealing (Kirkpatrick et al., 1983). The model pre-dates modern deep learning, and is similar to Naïve Bayes models. As such, the frequency and enrichment terms are calculated from the training sequences, but there is no test/train split or cross-validation metrics per se. Two models were produced, using either U6 promoters alone, or U6 and U3 promoters together. Novel expression elements generated by these models were screened for allergenicity and toxicity risks with bioinformatic tools before use (Codex_Alimentarius, 2009; To et al., 2021).

### Genome editing constructs

The CRISPR/Cas12a system from *Lachnospiraceae bacterium* ND2006 (LbCas12a; Zetsche et al., 2015) was used throughout this study. The LbCas12a coding region was codon optimized for maize (**Figure 1B**; **Supplementary Material S2**; Kouranov et al., 2022) and was flanked by two nuclear localization signals from the heat stress transcription factor 1 (HSFA1) gene of tomato (*Solanum lycopersicum* L.; Doring et al., 2000). For both protein-coding and non-coding transgenes, expression was conferred by a combination of gene expression elements that are collectively called a gene expression cassette. The Cas12a gene cassette was driven by a maize ubiquitin promoter (Zm.UbqM1; Christensen et al., 1992) and terminated by the 3’ untranslated region (UTR) of the rice lipid transfer protein (Os.LTP; Vignols et al., 1994). The crRNA cassettes for protoplast studies included the variable promoter sequences followed by a standard LbCas12a repeat (GAATTTCTACTAAGTGTAGAT), where the initial ‘G’ was added for proper expression by U6 promoters, a spacer corresponding to the genomic target sites, and a poly-T terminator (**Figure 1B**). In all constructs for for the multiplex editing of 30 targets, and for the constructs pM199, pM200, pM223, pM225, pM226, and pM230, all 23 bp spacer sequences were flanked with LbCas12a repeats on both sides. Sequences were either synthesized (BioBasic, Amherst, NY, USA; biobasic.com) or PCR amplified with Pol III promoters using Ultramer primers (IDT, Coralville, IA, USA; idtdna.com).

### Maize protoplast transformation

Protoplasts were isolated from leaves of etiolated seedlings of *Zea mays* cultivar 01DKD2. PEG-mediated protoplast transformation was previously described (Sheen, 2001). In each well, 0.8 pmol LbCas12a and 1.6 pmol crRNA plasmid DNA were co-delivered into approximately 320,000 protoplast cells. Two additional plasmids carrying Renilla and Firefly luciferase cassettes optimized for maize gene expression were included as transformation quality controls. Each transformation was replicated in four wells. The transformed protoplasts were incubated at room temperature in the dark for 48 h. Luminescence, indicative of protoplast transformation, was quantified in each treatment using Dual-Glo Luciferase Assay System (Promega, WI, USA; https://www.promega.com). Luminescence values were checked against control wells lacking transformed DNA within each experiment. For a treatment to pass this quality control step, the ratio of luciferase in the treatment compared to the no-DNA control needed to be at least 100 fold. Total genomic DNA was isolated from the entire cell suspension.

### Maize plant transformation

Mature seed embryo explants from the cultivar 01DKD2 were transformed via *Agrobacterium* as described previously (Ye et al., 2022). Briefly, binary vectors carrying the *epsps-cp4* selectable marker, *LbCas12a*, and various crRNA expression cassettes were transformed into a VirG constitutive *Agrobacterium* strain using gentamicin 30 mg/L and kanamycin 50 mg/L for selection. After co-culture of mature seed embryo explants with *Agrobacterium*, the explants were transferred to consecutive multiple bud induction media. Primary transformant (R0) plants were selected and regenerated on a hormone-free medium containing 25 µM glyphosate. Transgene copy numbers were determined by TaqMan-based qPCR using the Os.LTP terminator (Vignols et al., 1994) of the LbCas12a cassette as an assay template. Leaf tissues were collected from regenerated seedlings at the V1 growth stage. DNA isolation and copy number assay were performed as described previously (Kouranov et al., 2022).

### Target sites and sequence analysis

All genomic target sites used in this study are listed in **Supplementary Material S3**. Valuable edits for biotechnology products may require targeting low efficiency sites. Therefore, high- and low-efficiency target sites in genic and intergenic regions across a range of chromosomes were used to test a variety of editing conditions. Non-essential, non-linked sites across chromosomes were selected to test multiplexed editing of many independent locations simultaneously. Regions around the target sites were amplified using either protoplast or plant genomic DNA samples as templates. Amplicon sizes were kept below 200 bp to ensure that a paired-end workflow generating 2x150 bp sequences, would cover the entirety of the target site from at least one direction. The amplicons were sequenced by the Illumina HiSeq platform (San Diego, CA, USA; illumina.com). Reads were trimmed and mapped to the reference sequences as previously described (Rymarquis et al., 2024). The reads carrying insertions or deletions (indels) in the target sites were counted, which was used as an indirect measure for chromosome cleavage. For both protoplast suspensions and transgenic plants, the indel rates were determined by using the following formula: Indel% = 100 × reads carrying indels/total reads. In protoplasts, these individual edit percentages were averaged among the four replicates for each treatment. In plants, the above-calculated indel percentages per plant were used as an input to further calculate editing rates at the population level using two different methods. In the first approach, referred to as “average indel rate in sequencing reads” below, individual editing rates from leaf samples were averaged over the population of plants. In the second approach, we estimated the proportion of the population likely to transmit the edits to the next generation, which we termed “advanceable.” This estimate was based on the proportion of plants with an indel rate over a 10% threshold. Maize plants with edits over this threshold are more likely to have heritable edits (data not shown).

### crRNA expression studies

Leaf tissues were collected from regenerated seedlings at the V1 growth stage. Direct-zol RNA MiniPrep Kits (Zymo Research, Irvine, CA; https://www.zymoresearch.com) were used to extract total RNA according to manufacturer’s instructions. A subset of RNA samples was run on 5300 Fragment Analyzer System (Agilent, Santa Clara, CA; https://www.agilent.com) to confirm RNA quality (RNA Integrity Number > 7). Expression levels from computationally derived promoters were measured in two approaches, as detailed below.

In the multiplex editing experiment, the pre-crRNA species, each carrying multiple tandem spacers targeting different loci, were quantified. Total RNA was converted to first strand cDNA using a random primer mix in the High-Capacity cDNA Reverse Transcription Kit (Applied Biosystems, Foster City, CA; https://www.thermofisher.com). Real-time PCR reactions were done using TaqMan (TM) assays. The TM primers and probes assayed three consecutive spacers on each pre-crRNA species. A dilution series of pooled cDNA was used to confirm amplification efficiency and a no template control was used to confirm specificity. Expression values for the RNA of interest from each sample were normalized to the geometric means of the expression values of the housekeeping genes eIF1A and EF1a from that same sample.

When testing for increased crRNA expression targeting a single locus, first strand cDNAs were generated from processed, mature crRNAs using specific reverse transcription primers and Custom TaqMan Small RNA Assay kits (Applied Biosystems, Foster City, CA; https://www.thermofisher.com). Real-time PCR reactions were done using TM assays. The TM primers and probes assayed mature crRNAs. A dilution series of synthetic RNA was used to confirm amplification efficiency and a no template control was used to confirm specificity. Expression values for the RNA of interest from each sample were normalized to the geometric means of the expression values of the housekeeping genes eIF1A and EF1a from that same sample.

### Statistical analyses

#### Relative RNA expression

To compare RNA expression for each gene, Kruskal-Wallis rank sum tests were used to test for significantly different expression across all promoter-crRNA configurations. For CP4, there was no significantly different expression across the promoter-crRNA configurations. For Bmr3 and Cas12a, there was significantly different expression across the promoter-crRNA configurations (p < 0.001). For these two genes, therefore, pairwise comparisons of each configuration with the same number of crRNAs (e.g., 1PX8, 2PX4, and 4PX2) were performed using Wilcoxon rank sum exact tests.

#### Editing rates

To compare editing rates of interest, Kruskal-Wallis rank sum tests were used to show significantly different editing across treatments (p<0.001). Pairwise comparisons for individual treatments of interest were performed using Wilcoxon rank sum exact tests.

#### In-planta vs protoplast editing rate comparison

The mean indel rates for each construct were compared between protoplast and in-planta testing using a Spearman correlation test.

## Results

### Diverse monocot Pol III promoters enabled editing in maize protoplasts

Altogether 42 U6 promoters and 24 U3 promoters from maize and seven other monocotyledonous species were mined from public and Bayer proprietary databases as a training set for a proprietary generative machine learning model. A subset of 15 endogenous U6 promoters were directly tested (**Supplementary Material S1**). Three endogenous maize U6 promoters, one each from chromosomes 1, 2 and 3 (referenced below as Chr01, Chr02 and Chr03, respectively), were tested in different length variants (160 bp and 400 bp) for genome editing. A chimeric derivative between Chr08 and Chr01 U6 promoters (160 bp) was used as a positive control (**Figure 1A**) across several experiments in this investigation.

To establish a baseline for promoter performance, LbCas12a indel editing rates enabled by maize U6 promoters were tested in maize protoplasts. The promoters were mined from U6 genes on chromosomes 1, 2, and 3, along with a chimeric U6 promoter from chromosomes 1 and 8 (**Figure 1**; **Supplementary Materials S1**). Editing rates were evaluated at an intergenic (Zm.7.1b) and a genic (Zm.Bmr3_2691) site (**Supplementary Material S3**). The chimeric Chr08:Chr01 promoter was used as an internal positive control for subsequent experiments. The three endogenous promoters were tested in two length variants (**Supplementary Material S4**). Above-background chromosome cutting was detected with most promoter species. The Chr02 U6 promoter, however, resulted in low editing efficiency. For example, the Chr02 U6 160 bp variant resulted in 0.018 ± 0.013% indel (mean ± standard deviation) at the Zm.Bmr3_2691 site. Both length variants included all required U6 promoter elements (TATA box, USE, and MSP) for all four promoter species. While the two length variants performed comparably for both Chr01 and Chr02, the longer version (400 bp) of Chr03 performed significantly better than its shorter counterpart (160 bp) for the Zm.7.1b target site (2.01 ± 0.60% indel compared to 0.93 ± 0.23% indel; Kruskal-Wallis rank sum test followed by Wilcoxon rank sum exact test, p < 0.05).

Twelve U6 promoters from six monocots other than maize were tested in the same protoplast system. Editing rates were evaluated at an intergenic (Zm.7.1c) and a genic (Zm.Bmr3_2691) target site (**Supplementary Material S5**). Most promoters performed comparably with maize Chr08:Chr01. However, Et_3478 from *Eragrostis tef* showed low performance for both Zm.7.1c (2.54 ± 1.32% indel) and Zm.Bmr3_2691 (0.051 ± 0.102% indel) target sites. Additionally, So_1047 from *Saccharum officinarum* showed low performance for Zm.7.1c (8.15 ± 1.25% indel).

### Computationally derived Pol III promoters enabled editing in maize protoplasts

**Figure 2:**
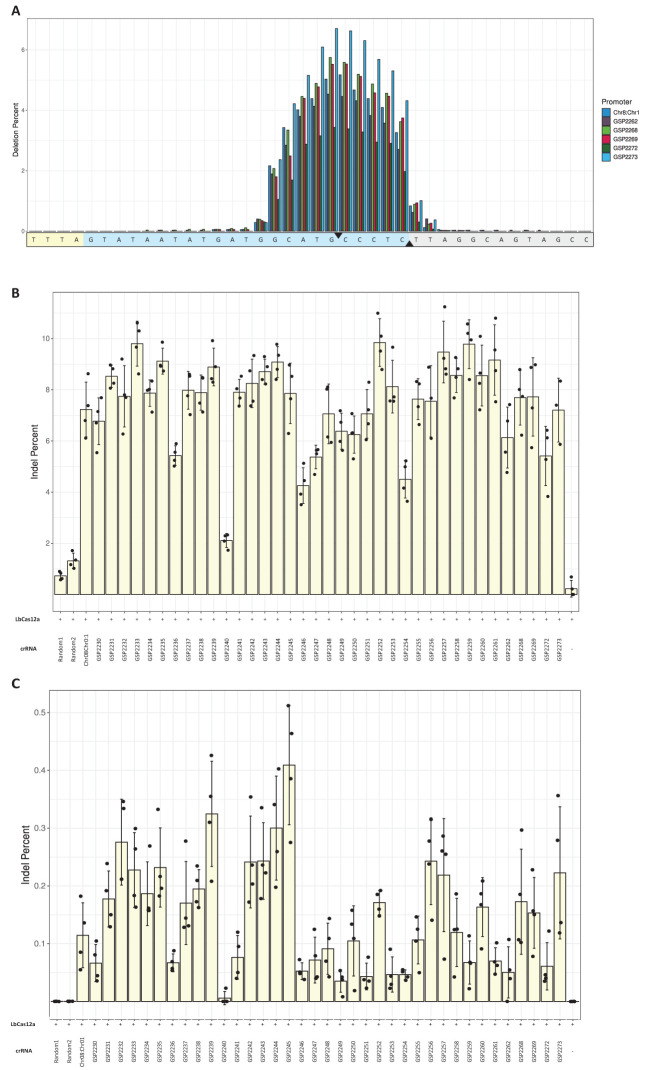
Novel Pol III promoters enabled CRISPR/LbCas12a editing. A chimeric maize U6 promoter (Chr08:Chr01) was used as a positive control. **(A)** Distribution of deleted nucleotides along the intergenic Zm.7.1c target site for five arbitrarily selected promoters (GSP2262, GSP2268, GSP2269, GSP2272 and GSP2273) and the positive control. Average deletion percentages among four replicates were calculated for each nucleotide position in a 40-bp region including the target site. The yellow- and blue-shaded portions of the sequence are the PAM and target site, respectively. The black triangles denote the nick sites of LbCas12a. **(B)** Editing rates at the intergenic Zm.7.1c target site. Two random, 300bp sequences were also used as negative controls. Bars are averages of four replicates (dots); error bars are standard deviations. **(C)** Editing rates at the genic Zm.Bmr3_2691 target site.

Thirty-seven novel 500 bp Pol III promoters were computationally derived using a proprietary generative machine learning model (To et al., 2021; Davis and Elich, 2025; **Supplementary Material S6**). Editing rates were evaluated at an intergenic (Zm.7.1c), and a genic (Zm.Bmr3_2691) target site in protoplast assays (**Figure 2**). In this experiment, most promoters were trimmed to between 280 and 300 bp in length. Trimmed promoters generally showed the same activity as the original 500 bp designs (**Supplementary Material S7**). Distributions of deletions along target sites were compared among a subset of the novel Pol III promoters (**Figure 2A**). Eight to ten base pairs around the upstream nick site on the non-target strand were deleted most frequently for all promoters tested. Editing rates were compared to a Chr08:Chr01 U6 positive control, a negative control lacking a crRNA cassette, and two 300 bp randomized sequences. A promoter was counted as active if it performed at or above the lower bound of standard deviation for the Chr08:Chr01 U6 (**Figures 2B, C**). In total, 27 promoters were counted as active for both the genic and intergenic sites. Thus, the hit rate for derivation of novel crRNA promoters was 73%. The distributions of editing rates were similar between promoters derived from a training set of U6 promoters and those derived from both U6 and U3 promoters (**Supplementary Material S8**).

### Computationally derived Pol III promoters enabled editing in maize plants

Nine promoters, representing a broad range of activities in the protoplast results (**Figure 2**), along with the chimeric Chr08:Chr01 control, were selected for *in planta* genome editing at the Zm.7.1c target site of maize (**Table 1**). Leaf samples from R0 plants with novel crRNA promoters showed averages of 41.8-76.1% indels, as measured by sequencing reads. A plant was counted as “advanceable” to the next generation if more than 10% of the sequencing reads from a leaf sample showed insertion or deletion. Between 65.5 and 100% of plants with novel crRNA promoters were advanceable.

**Table 1:** Editing rates at the Zm.7.1c intergenic target site in R0 maize plants in which the crRNA transcription was driven by novel Pol III promoters or the chimeric maize Chr08:Chr01 control.

Promoter	Counts of R0 plants	Average indel rate in sequencing reads	Counts of advanceable (>10% indel reads) R0 plants	Advanceable R0 plants rate
Chr08:Chr01	33	52.9%	25	75.8%
GSP2233	47	66.2%	38	80.9%
GSP2239	34	63.0%	29	85.3%
GSP2244	51	61.5%	44	86.3%
GSP2245	37	61.5%	28	75.7%
GSP2262	48	45.8%	35	72.9%
GSP2268	56	57.2%	46	82.1%
GSP2269	34	56.7%	27	79.4%
GSP2272	29	41.8%	19	65.5%
GSP2273	28	76.1%	28	100.0%

Only plants that carried one or two transgene copies of the transgenic editing machinery were analyzed.

The editing rates determined in protoplast testing (**Figure 2**) were compared to the *in planta* editing rates (**Table 1**) to test correlation (**Supplementary Material S9**). Of the two *in planta* editing calculation methods, the “average indel rate in sequencing reads” approach is more similar to the one used in the protoplast system. Therefore, we used this method and found a significant correlation between protoplast and *in planta* rates (Spearman correlation = 0.68, p < 0.05).

### Computationally derived Pol III promoters enabled multiplexed genome editing in maize plants

**Figure 3:**
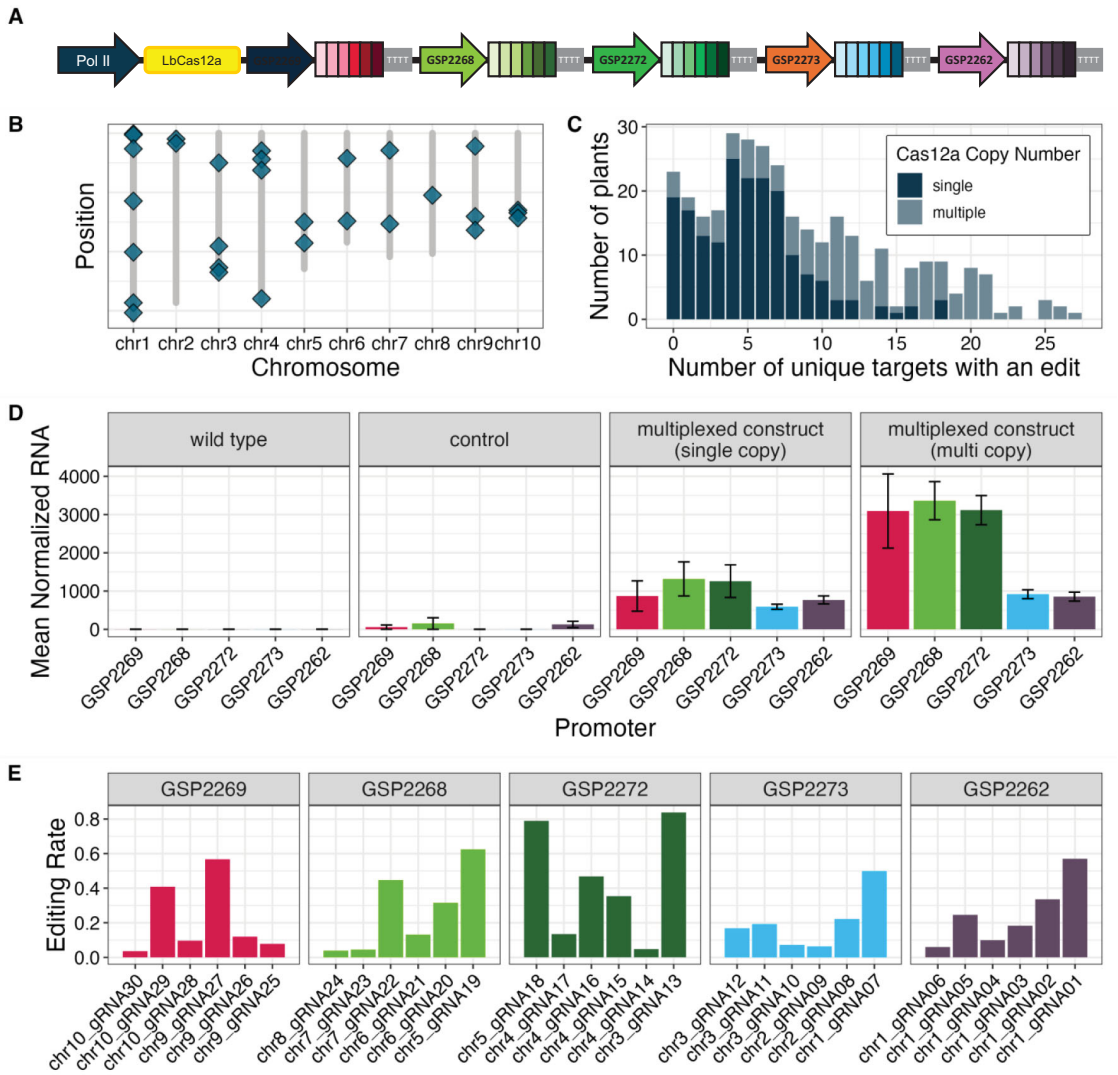
Novel Pol III promoters enabled multiplexed editing in maize plants. **(A)** Vector configuration for five computationally derived Pol III promoters used to drive expression of cassettes with six unique crRNAs each. **(B)** Graphical representation of relative positions for 30 unique crRNA targets (blue diamonds) in the maize genome. **(C)** The number of unique target sites with an edit across 330 assayed plants when a single copy (dark blue) or multiple copies (light blue) of the LbCas12a transgene were present in the assayed plant. **(D)** Expression of pre-crRNA species, represented as mean normalized RNA accumulation, from cassettes driven by novel Pol III promoters. Plants were transformed with an empty control vector (second panel, n=9 independent transgenic events) or the test vector present in either a single copy (third panel, n=34 independent transgenic events) or multiple copies (fourth panel, n=29 independent transgenic events). Error bars are standard error of the means. **(E)** Editing rates for each of the 30 unique crRNAs grouped by cassette promoter, calculated as the number of plants with an indel at the corresponding target site divided by the total number of assayed plants.

Five computationally derived Pol III promoters were used to drive the expression of five crRNA cassettes, each containing six unique spacer sequences, to target a total of 30 distinct genomic regions interspersed across all 10 maize chromosomes (**Supplementary Material S3**, **Figures 3A–C**). Editing was observed at all 30 target sites among the 333 R0 plants that were transformed with this construct. Within a single plant, up to 27 sites were edited at an advanceable rate (>10% indel reads) when multiple copies of the transgene were present and up to 18 sites were edited at an advanceable rate when only a single copy of the transgene was present (**Figure 3C**). The expression of each unprocessed crRNA transcript was measured using RNA Taqman assays (**Figure 3D**). The editing rate for individual crRNAs ranged from 3.6% to 83.8% (**Figure 3E**). Expression was compared to a control construct containing LbCas12a and no crRNA cassettes. All five of the computationally derived Pol III promoters showed expression, although the levels varied.

### crRNA cassettes driven by diverse Pol III promoters can be stacked to increase crRNA expression and editing rates

**Figure 4:**
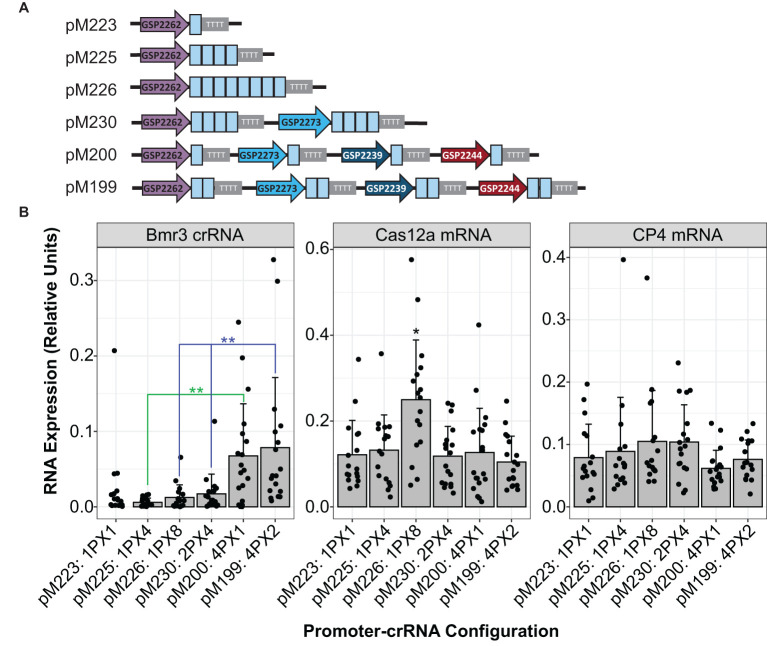
Stacking multiple crRNA cassettes driven by unique Pol III promoters increased accumulation of mature crRNA in maize plants. **(A)** Configuration of crRNA cassettes in constructs. The Zm.Bmr3_2691 spacer sequences are depicted as blue boxes. **(B)** Comparison across constructs of RNA expression of the mature crRNA targeting Zm.Bmr3_2691, LbCas12a mRNA, or CP4 selectable marker mRNA. The number of cassettes and number of spacers in each are indicated. For example, 4PX1 means four separate cassettes were in the construct with a single spacer each. Each data point represents expression from an independent transgenic event, at least 16 per construct. Significantly higher expression is marked with * or ** (Kruskal-Wallis rank sum test followed by Wilcoxon rank sum exact tests, p < 0.05 or 0.01, respectively).

To improve low editing rates, the same spacer was tested in various multiplex crRNA configurations. The spacer for the low-efficiency Zm.Bmr3_2691 target site was repeated multiple times in the same crRNA array driven by one promoter. This was compared to a configuration in which the same spacer was placed in separate cassettes driven by diverse Pol III promoters (**Figure 4A**). Multiple spacers in a single array did not improve editing rates in plants as compared to their singleton counterparts (**Table 2**). However, stacking multiple crRNA cassettes resulted in a significant increase in mature crRNA accumulation (Kruskal-Wallis rank sum test followed by Wilcoxon rank sum exact tests, p < 0.01, as detailed in **Figure 4B**) and increased the rate of advanceable edited plants from 0% to 10.4% (**Table 2**). Selectable marker gene RNA expression levels, used as a control, were constant across constructs (**Figure 4B**). A construct with multiple spacers in a single array (pM226) did show significantly higher LbCas12a RNA expression but did not result in an increased editing rate or crRNA accumulation (Kruskal-Wallis rank sum test followed by Wilcoxon rank sum exact tests, p < 0.05).

**Table 2:** Comparing various crRNA configurations along with a single LbCas12a cassette at a genic target site (Zm.Bmr3_2691) for chromosome cutting activity in maize plants.

crRNA Configuration	Counts of R0 plants	Average indel rate in sequencing reads	Counts of advanceable (>10% indel reads) R0 plants	Advanceable R0 plants rate
pM223_1PX1	56	0.2%	0	0.0%
pM225_1PX4	52	0.0%	0	0.0%
pM226_1PX8	59	0.0%	0	0.0%
pM230_2PX4	57	0.3%	0	0.0%
pM200_4PX1	48	5.9%	5	10.4%
pM199_4PX2	51	5.6%	4	7.8%

The number of cassettes and number of spacers in each are indicated (see also **Figure 4**). For example, 4PX1 means four separate cassettes were in the construct with a single spacer each.

**Figure 5:**
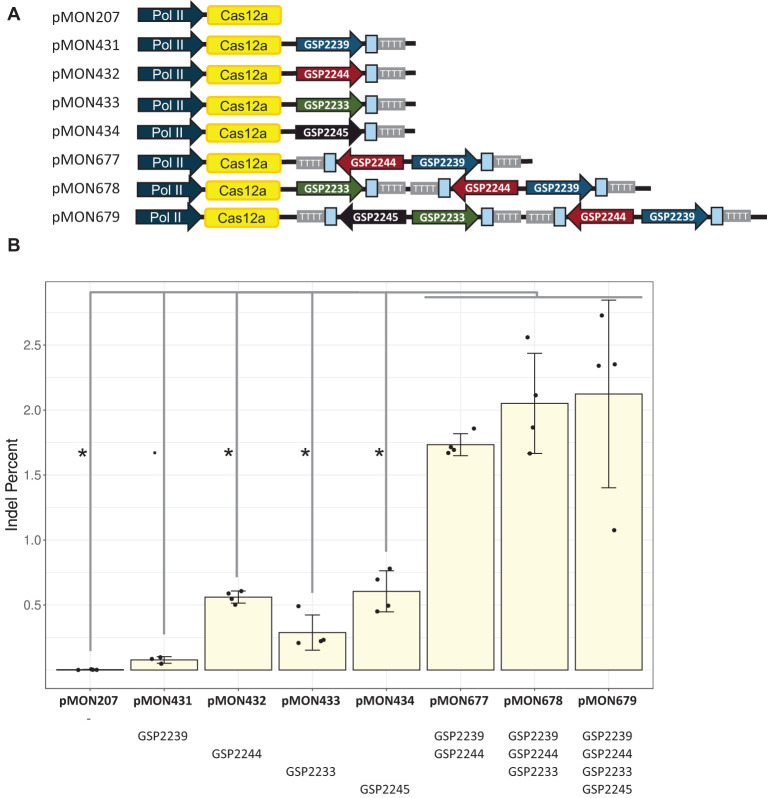
Stacking multiple crRNA cassettes driven by unique Pol III promoters improved editing efficiency at the low efficiency genic target site (Zm.Bmr3_2691) in maize protoplasts. **(A)** Diagram of plasmids with different numbers and configurations of crRNA cassettes, each with a single LbCas12a cassette. The Zm.Bmr3_2691 spacer sequences are depicted as blue boxes. **(B)** Editing rates enabled by these configurations. The bars represent averages of four replicates (dots); error bars are standard deviations. Significant differences are marked with * or. (Kruskal-Wallis rank sum test followed by Wilcoxon rank sum exact tests, p < 0.05 or 0.06, respectively).

In similar experiments conducted in protoplasts (**Figure 5**) and plants (**Table 3**), single-spacer cassettes driven by five computationally derived promoters were placed as singletons or in multiplex in the same constructs along with a single LbCas12a cassette as depicted in **Figure 5A**. The same trend was observed: using multiple Pol III cassettes increased editing rates (**Figure 5**, **Table 3**). Specifically, the double, triple and quadruple constructs all enabled significantly higher editing than their singleton counterparts (Kruskal-Wallis rank sum test followed by Wilcoxon rank sum exact tests, p < 0.05 or 0.06, as detailed in **Figure 5B**). For the Zm.Bmr3_2691 target site, the double construct (pMON677) enabled 1.73 ± 0.08% indel, the triple construct (pMON678) enabled 2.05 ± 0.39% indel, and the quadruple construct enabled 2.12 ± 0.72% indel. In contrast, the most efficient singleton construct (pMON434), enabled 0.61 ± 0.16% indel.

**Table 3:** Improvement of low editing efficiency at a genic target site (Zm.Bmr3_2691) in maize plants by using multiple crRNA cassettes along with a single LbCas12a cassette.

crRNA Configuration	Counts of R0 plants	Average indel rate in sequencing reads	Counts of advanceable (>10% indel reads) R0 plants	Advanceable R0 plants rate
pMON435_1PX1	38	2.8%	1	2.6%
pMON431_1PX1	34	2.3%	2	5.9%
pMON432_1PX1	41	4.2%	3	7.3%
pMON433_1PX1	33	7.7%	4	12.1%
pMON434_1PX1	27	8.1%	3	11.1%
pMON677_2PX1	35	8.1%	5	14.3%
pMON678_3PX1	50	11.5%	10	20.0%
pMON679_4PX1	34	17.5%	7	20.6%

The number of cassettes and number of spacers are indicated (see also **Figure 5**). For example, 4PX1 means four separate cassettes were in the construct with a single spacer each. pMON435_1PX1, also included in this experiment, consisted of the single LbCas12a cassette as well as a single crRNA cassette driven by the Chr08:Chr01 U6 promoter.

Results from protoplasts showed that the addition of the second crRNA cassette contributed the most to editing rates. The GSP2239-driven cassette plus GSP2244-driven cassette was more than 20 fold more efficient than GSP2239-driven cassette alone, and more than 3 fold more efficient than GSP2244-driven cassette alone. The improvements diminished with the third and then the fourth added cassettes. For example, the triple was about 1.2 fold more efficient than the double. In contrast, the efficiency gains from these same constructs in generating advanceable plants were more steady. The GSP2239-driven cassette plus GSP2244-driven cassette was about 2 fold more efficient than GSP2239-driven cassette or the GSP2244-driven cassette alone. The triple was about 1.4 fold more efficient than the double.

## Discussion

This study developed novel, computationally derived Pol III promoters for crRNA expression. Using an algorithm previously developed for novel Pol II elements, we derived 37 novel Pol III promoters. Most of these novel promoters enabled editing rates similar to a control U6 promoter. These editing efficiencies were demonstrated in both maize protoplast systems and plants. However, 27% of the novel promoters enabled editing below our “hit” threshold in protoplasts, and the advanceable plant rate was only 65.5% for GSP2272. The observed variation in editing performance from the novel pol III promoters may be due to differences in transcription factor binding site efficacy and/or location, which in turn could lead to differences in crRNA accumulation and editing efficiency.

The Pol III promoters of monocot U6 and U3 snRNAs include three conserved elements: TATA box, upstream sequence, and MSP elements (Waibel and Filipowicz, 1990; Marshallsay et al., 1992). While the first two have conserved sequence and are located within 60 bp of the TSS, MSP elements tend to vary in number, position, and sequence (Connelly et al., 1994). The shorter versions (~300 bp) of the novel promoters generally performed just as well as the longer versions (500 bp). But the longer version (400 bp) of Chr03 performed significantly better than its shorter counterpart (160 bp) for the Zm.7.1b target site. This promoter may harbor other important elements in more distal positions, such as additional degenerate MSP elements. While most non-maize monocot U6 promoters performed comparably with the maize Chr08:Chr01 control, Et_3478 and So_1047 showed low performance. This may be due to sequence divergence between these promoters and maize Pol III promoters and/or lack of maize-specific transcription factor binding sites. Future experiments could use mutation to directly test the importance of putative transcription factor binding sites in both proximal and distal locations.

Maize protoplasts were used to test gene editing system variables in this study. Protoplasts are generally recognized as a valuable genome editing platform to accelerate learning cycles prior to expensive and time-consuming *in planta* transformations (Feng et al., 2016; Weiss et al., 2020; Fierlej et al., 2022; Gaillochet et al., 2023). There was a significantly positive correlation between editing rates in protoplasts and plants in this study (Spearman correlation = 0.68, p < 0.05). This correlation was modest. There were also differences between plants and protoplasts in the rate of efficiency gains when adding crRNA cassettes. These discrepancies are potentially due to differences in chromatin context or tissue-specific factors between the testing systems. Future experiments could test the latter directly through protoplasts from multiple tissue types.

One application of having many effective Pol III promoters is to target multiple unique genomic locations simultaneously. Cas12a in particular lends itself to multiplex editing, as it requires only a crRNA transcript and multiple spacers can be expressed in a single cassette. Multiplex editing has been enabled through various approaches, including stacking up to 25 spacers (Campa et al., 2019; Zhang et al., 2021). The largest arrays require complex cloning and rely on Pol II rather than Pol III promoters, as these naturally express longer transcripts. Alternatively, the ability to multiplex crRNA cassettes, each with a unique Pol III promoter and a smaller number of spacers will reduce element redundancy, facilitating DNA synthesis and cloning. Computational derivation of Pol III promoters enables this modular cassette stacking, as demonstrated here through multiplexing five crRNA cassettes containing six spacers each targeting a total of 30 sites. Each set of spacers showed a range of editing efficiencies. While crRNA expression is necessary for editing, the sequence and epigenetic features of a target site may contribute more to overall editing efficiency.

In plants, a subset of target sites, while including compatible protospacer-adjacent motifs (PAMs) for a CRISPR system, are nevertheless refractory to chromosome cutting (Weiss et al., 2022; Zhong et al., 2018). The Zm.Bmr3_2691 target site in this study is an example of a low efficiency editing site. Adding multiple crRNA cassettes using different promoters but targeting the same genomic site mitigated this lower efficiency. Taken together, protoplast and plant results across multiple experiments demonstrate that editing rates can be increased by additional cassettes driven by the novel promoters GSP2239 and GSP2244 in either head-to-head or head-to-tail orientations. Future experiments could test additional combinations of crRNA cassettes to better understand and predict how to increase efficiency. Intriguingly, a crRNA cassette in which a single promoter drove an array of the same spacer sequence in tandem did not produce the same results. The observed differences in editing and mature crRNA accumulation may be due to separate expression cassettes increasing crRNA transcription, processing efficiency, and/or stability.

These novel Pol III promoters increased genome editing flexibility and efficiency in maize through both allowing multiplexing of unique spacers targeting distinct sites as well as expressing multiple copies of the same spacer to increase cutting efficiency. Future experiments could test if these approaches can be extended to other crops, such as soybeans. While these novel promoters were tested with the CRISPR/LbCas12a editing machinery in the current study, they could also be useful in other transgenic systems requiring heterologous nuclear expression of non-coding RNA species. These include commonly used genome editing systems, such as CRISPR/Cas9, as well as gene silencing systems expressing small interfering RNA (siRNA) or short hairpin RNA (shRNA; Ma et al., 2014).

## Data Availability

The original contributions presented in the study are included in the article/**Supplementary Material**, further inquiries can be directed to the corresponding author.
