# Supplementary material for: Computationally derived RNA polymerase III promoters enable maize genome editing
Source: Front Plant Sci. 2025 Mar 19;16:1540425. doi: 10.3389/fpls.2025.1540425 (PMC11961915; doi:10.3389/fpls.2025.1540425)
Supplement: Supplementary file 1 [file DataSheet1.docx]

Nagy et al. Supplementary Material

**Supplementary Material S1 |** U6 promoters from seven monocotyledonous species tested in this study. The species of origin are abbreviated as follows: *Zea mays* (Zm), *Eragrostis tef* (Et), *Oryza sativa* (Os), *Setaria italica* (Si), *Sorghum bicolor* (Sb), *Saccharum officinarum* (So), *Brachypodium distachyon* (Bd).

>Zm_Chr01_U6

AGGAAAAGAAGAGGTGATTACTGTACCTATTCGTCTTTGTATCGGAATATAAATTTATCACTATTTTATGATAAAGTAAATCTGTTTCCCTGTAGAGTTAATTAATTAATGTAAGTATAAGCGTAATTTATAGGGCACTAGTAGGACTGTCGACTGTGCGCTCGGCCCGGATAATGCGTCAAAAGCGAAGACGTGCACGTGGGATGGGAAAACACGAAGCGTGGTCTGCTTTTTCGCATGATATCTGGGCCGCACCAAAGAATCCAGCCCACGCGGCGTGGCGCCGTCGTTACGGCTTGCGGGGGAAGGAAACGAGGGACGAACCGAGATTTAGCACCAGACCGGCCAGCGAGCATTGCAGACACCGGCTTATAAGTTCAGCTGCGACTACCACTCC

>Zm_Chr02_U6

TTATATGTTACCGTTGCAAAGCACGGGCACTCACCTAGTATATAATATAACATCAGTCGTACGTAATGTACTGATGGGCGGGTTAACAAATGTCACTCACTATCAGCACCAGCAGCGCTTAGATGCATCCGGCCGGGCCAAGACCCAGGACCAGAAAGCGCGCACGTTCACAGCGGATGCTGATGGGTTAGATCGACTGATCGAGGAAGAGGAGAGCTTAATTAAGAAACGCCCTGTTCCGCTTTGCTAGCTTGCGCCCTGACTGTCCAGCCCACGCGCTTCGGTCCGATTCACATGCTAGGCTGGTGCAAGCGAGCCGAGACTTTTTTTTAGAACCACCTTGCTCAGCAAACCTTAGGAACACCGGCTTATAAGTCGAAGCGAAGCGCTGTGCACT

>Zm_Chr03_U6

GGATTCGGTGTTCTTTATTAGGTTCTCGCCGAATATGGTTCTCAGTTATGACCTAACGGTGTCCACAAGAGTTCGCCAGGATTTATACAACTATTTTCTTATTTATTTCTTTAACATTTTCCCTTCTACGCACAATAGGAGATAATGTCAAGCGTTGACGGTGCACATATATTTGTTTTTTTAAAGGCGTAGTGGCGTGTGTGCAAAAACATCCTCACAGGAAAGACACGAAGAAACATGGTCAATGGCCCATTATATAAAGCACCGCCACAAAGCCCAAATACCAGTTCGTCGGTGGAGCAAGTAACGCGCTAGGCAACAGGCAAACAGTTTGTCCCACCTCGTCCAGTCACAAAGGCAAAGCGTGACTTATAAGCCAGAGCGGAAGAACCATACC

>Et_5287_U6

TAACATGTGAACTGTGCATTGCCGTACATCGTACATGTATAATATATTCTGTAGCATGTACACAGAATAGTCAGAACCGAGAAAAAAGACGCGGTGCAAGGAGCCCTAGTCGGGCCGTAGAAGCGTTAAACCCACACGGGCCTCAACAGGCGGGCTGTGGGCCGTGTACGCCAGATCGCGCGCGAGGGGACCCGTGAGAAGTGGACGGTATTGGTAGCGGGTACAGCCAGCGTTTAGTACCACATGGCTAAACTAAACACATGTGAGCTACCTTATAACCTGAAGCCCAACAGCCGTGGT

>Et_3478_U6

GTAATGTTCGTACGGTTTTTCTTTTTACTTTTTTGACATTTCGAAGCGTTCATGCGTGTGTGCGTTCTAAATTCTAATGTTACACAGCAAACAGGGCGTGAAAGCAAAGAAAAGATGTACAACTCGGTTTAATGGGCCGTGGTTTTATCGAGCGCTCGCTTCAGTTTTATACTGGGCCGGCCCACGAGAGCGTGACAGGCGCGTGAACTAAATCGGGAACTGCCGTACCAGGTTTAGTCCCCCAACGGAAATTAAGAGAGGTGTAAGCAAGCTTATAATCTGAGGCGCTGAGACTGCCTT

>Os_4793_U6

GTTCGTGCAGACAGAGCTCATACTTGACTACTTGAGCGATTACAGGCGAAAGTGTGAAACGCATGTGATGTGGGCTGGGAGGAGGAGAATATATACTAATGGGCCGTATCCTGATTTGGGCTGCGTCGGAAGGTGCAGCCCACGCGCGCCGTACCGCGCGGGTGGCGCTGCTACCCACTTTAGTCCGTTGGATGGGGATCCGATGGTTTGCGCGGTGGCGTTGCGGGGGATGTTTAGTACCACATCGGAAACCGAAAGACGATGGAACCAGCTTATAAACCCGCGCGCTGTAGTCAGCTT

>Os_1741_U6

AGGAGCACATATACAAACCGGTTTTATTCATGAATGGTCACGATGGATGATGGGGCTCAGACTTGAGCTACGAGGCCGCAGGCGAGAGAAGCCTAGTGTGCTCTCTGCTTGTTTGGGCCGTAACGGAGGATACGGCCGACGAGCGTGTACTACCGCGCGGGATGCCGCTGGGCGCTGCGGGGGCCGTTGGATGGGGATCGGTGGGTCGCGGGAGCGTTGAGGGGAGACAGGTTTAGTACCACCTCGCCTACCGAACAATGAAGAACCCACCTTATAACCCCGCGCGCTGCCGCTTGTGTT

>Si_3506_U6

CTACATGGATCGTCTCCATCTTTTACTTACCAGCCCAACAATGAATGCGTCTGGCCCAGCCCAACAACGCGTACAAGGCCCCAGCACGGAAAATTAGTGGACTGGGACGAAGAAGCCCCGGATGGCTCCGTTTCGAGAGACTGGGCTAAAGCGTGCGACCGAGCGCGGCGGGCGGTTTGGGCGCTTCCGCCTTCGCGCTTGGGTCCGTGTCGTCGCCCATTGCGCAGCAGAGTTTAGTACCACCTCGTCCAGATAATGACGGAAACGCCAGGTTATAATCCGAAGCGTTGCAGCAGCGTT

>Si_3591_U6

AGGTTCCATAAACCTTCCATTGCTGTCTCTGGTTCGCAGCCATCAGTATCCTGTACAGAATACGTTCATAGTAATATCATACATATGCTGAACACTTCTTGGACAGCACGGCCAGCCCGTGGGCCGCGGGCCGTAAGCGGCAGCTCACACGCGGGCCGGATCCGTCGGGTGCGGGAGCGGACAGCACGGCTGCACGGGAACGAGCAAGCGGAACACCGGAAGCAGAGCCAATGTTAGTACCGTACCGCCAAGCCAAGGACGCGCAGACCAAGTTATAAACTGAGCCGCGGAGACGATACT

>Sb_0550_U6

TTTTTTTAGGAAATCAGAATTCTTCTGTGATGACTCGAAACGCCAGATGCATCCGGCCCAGCTAAGCGCGCACGTACACAGCGTGTGTGCACGCCAGATTTTGAGGGTTGGTCGAGGGAGAGGCCGCTTTCAAAGGTTGGGCCCCGATTGTCCAGCCCACGGAAGTGCGGCCCAACTGGAGTCAGCCGTCGAGCGCGCGCTTCGGTCCGTCTCAGTTCGGTGCGAGCAGAGTTTTAGCACCACCTCGCCCAGCCAACTTGAGGAACGCCAGCTTATAAGCTCAGGCGTAGCCACAGCGTC

>Sb_1567_U6

AAGATTTATACAACTTTTTTCTTATTTATTTCTGGAACTTCTACGTACAGTAGGAGATAGTGTCACGGTGCTGACGGTGGGTCGCTGTATATTAGTGGCGTATGTGCCAACACAGAAAGGCCACGGAGAAACATGGGCCATGGCCCATGGACCAATACGAAGGACCGCAACGAAGCCCAAACAGCAGTTCGTCGGTGCTCGGTGAAGCAACGCGCTGAGCAACAGGCAATCGTTTTGTCCCACCTCGGCTAGTCACGGAGGGAGAACGTAGCTTATAAGCCGAGGCGCAGGCACCGCATC

>So_1047_U6

TTGATTTAGGAAAGAGCTCCATAAAACACCATATTAGTGTGCACCCGCACCGTCGTCATCTCTTCTTCCTCCCATCTCGTGAATAAAAATACCTAGAGACATATGTATCAGGGCCCATATCGATCTCCCAACTAAATGGGCCATTGGAAAGCAAGCAGCCCACACGCAGCTCTTAGGTCTGTCAGCATCAAATTATGGAGCAGCATTGCGGGAGGCAACTAATGATGTTTAATTAATGCCACCTCGCTAGCGCAATGCGAGAAAGCTTTGCTTATAAGCTGTTGTGCATAGCTTAATCTT

>So_2760_U6

GAGAGGCGCACTTATCATGACTCGGAGTACCCTGGCAGACCTGCTAATTGTACACTTAATGCGTCAAAGTGCAAAAGGGCCTGCAATCTTCCCAAGGGCAAATACGAAGCCCACTTTGTTGTTCCCCTGATAGCTGGGCCGTGCTAAACGATGACACGCATAAATCGAGAAATGGTCGAAAAGGCATCAGCACCATGGCTTGCAGGGAAGGGAACGAGCGACCAACTCGGGATTTAGTACCAAACCGGCCAGCGAACGACCCAAACGACAGCTTATAAGCCGAGCTGTGGTAACTGCAAT

>Bd_5436_U6

TGTTCGTTCCAATTTCCAAAGGCTGAATTGCCATTTTAGCACCGCGTGGAAAACATAGACGCTTGGCTTCATTACTCTTTTTTTCCCAATTTGAAGGAAAGCTCGCTTCATTAATTGGGCCAGCAGGTGGGGAATATACATGGCCCACGTGCACATTCGGCCTGCTTATTGTTAGACTGCAGCCCGCTCCCGCGACTTCGTGGCGCTTAACGTGTGCAGCAGAGGACCCTCGTTTAGTCCCACATCGCCCATCGTGAAAGATCCAGACCTGTTTATAAACAGAGCGCTAGCTCCTGCCTC

>Bd_8680_U6

GAAGATTGGGGATAAGAGGAGGGGGTTTGGCGGGAAGCCTGGGGGGCGGCGTGGCGGGAAGCTTAAAAGACTGGGTAGCGAAAAGCCTGGAAGAAATAAGTCGCAGGTTAGTTCAGCCCACTTCGTTAAGTTGGGCCGAGATGCGGGAAGGAAACGCGGTTGTGGCCCAGCTCTCGTTTCGAAAGCGTCCTGCGGACTCCGGGCGCTTGCCGCGCGCAGTGGAGAACTTGTGTTTAGTCCCACATCGGCAAACGACAGTGGTGCCAAGCAGTTTATAAACAGAGCGCTAGCTACTGTTTC

**Supplementary Material S2 |** Expression elements and coding regions of the LbCas12a cassette.

>Promoter - Zm.UbqM1

GTCGTGCCCCTCTCTAGAGATAAAGAGCATTGCATGTCTAAAGTATAAAAAATTACCACATATTTTTTTGTCACACTTATTTGAAGTGTAGTTTATCTATCTCTATACATATATTTAAACTTCACTCTACAAATAATATAGTCTATAATACTAAAATAATATTAGTGTTTTAGAGGATCATATAAATAAACTGCTAGACATGGTCTAAAGGATAATTGAATATTTTGACAATCTACAGTTTTATCTTTTTAGTGTGCATGTGATCTCTCTGTTTTTTTTGCAAATAGCTTGACCTATATAATACTTCATCCATTTTATTAGTACATCCATTTAGGATTTAGGGTTGATGGTTTCTATAGACTAATTTTTAGTACATCCATTTTATTCTTTTTAGTCTCTAAATTTTTTAAAACTAAAACTCTATTTTAGTTTTTTATTTAATAATTTAGATATAAAATGAAATAAAATAAATTGACTACAAATAAAACAAATACCCTTTAAGAAATAAAAAAACTAAGCAAACATTTTTCTTGTTTCGAGTAGATAATGACAGGCTGTTCAACGCCGTCGACGAGTCTAACGGACACCAACCAGCGAACCAGCAGCGTCGCGTCGGGCCAAGCGAAGCAGACGGCACGGCATCTCTGTAGCTGCCTCTGGACCCCTCTCGAGAGTTCCGCTCCACCGTTGGACTTGCTCCGCTGTCGGCATCCAGAAATTGCGTGGCGGAGCGGCAGACGTGAGGCGGCACGGCAGGCGGCCTCTTCCTCCTCTCACGGCACCGGCAGCTACGGGGGATTCCTTTCCCACCGCTCCTTCGCTTTCCCTTCCTCGCCCGCCGTAATAAATAGACACCCCCTCCACACCCTCTTTCCCC

>Leader - Zm.UbqM1

AACCTCGTGTTCGTTCGGAGCGCACACACACGCAACCAGATCTCCCCCAAATCCAGCCGTCGGCACCTCCGCTTCAAG

>Intron - Zm.UbqM1

GTACGCCGCTCATCCTCCCCCCCCCCCTCTCTCTACCTTCTCTAGATCGGCGATCCGGTCCATGGTTAGGGCCCGGTAGTTCTACTTCTGTTCATGTTTGTGTTAGAGCAAACATGTTCATGTTCATGTTTGTGATGATGTGGTCTGGTTGGGCGGTCGTTCTAGATCGGAGTAGGATACTGTTTCAAGCTACCTGGTGGATTTATTAATTTTGTATCTGTATGTGTGTGCCATACATCTTCATAGTTACGAGTTTAAGATGATGGATGGAAATATCGATCTAGGATAGGTATACATGTTGATGCGGGTTTTACTGATGCATATACAGAGATGCTTTTTTTCTCGCTTGGTTGTGATGATATGGTCTGGTTGGGCGGTCGTTCTAGATCGGAGTAGAATACTGTTTCAAACTACCTGGTGGATTTATTAAAGGATAAAGGGTCGTTCTAGATCGGAGTAGAATACTGTTTCAAACTACCTGGTGGATTTATTAAAGGATCTGTATGTATGTGCCTACATCTTCATAGTTACGAGTTTAAGATGATGGATGGAAATATCGATCTAGGATAGGTATACATGTTGATGCGGGTTTTACTGATGCATATACAGAGATGCTTTTTTTCGCTTGGTTGTGATGATGTGGTCTGGTTGGGCGGTCGTTCTAGATCGGAGTAGAATACTGTTTCAAACTACCTGGTGGATTTATTAATTTTGTATCTTTATGTGTGTGCCATACATCTTCATAGTTACGAGTTTAAGATGATGGATGGAAATATTGATCTAGGATAGGTATACATGTTGATGTGGGTTTTACTGATGCATATACATGATGGCATATGCGGCATCTATTCATATGCTCTAACCTTGAGTACCTATCTATTATAATAAACAAGTATGTTTTATAATTATTTTGATCTTGATATACTTGGATGATGGCATATGCAGCAGCTATATGTGGATTTTTTAGCCCTGCCTTCATACGCTATTTATTTGCTTGGTACTGTTTCTTTTGTCCGATGCTCACCCTGTTGTTTGGTGATACTTCTGCAGGTC

>Nuclear Localization Signal - Le.HSFA1

GGATCTAAGAAGAGAAGAATTAAACAAGAT

>Coding Region - LbCas12a

ATGTCGAAGCTCGAGAAGTTCACCAACTGCTACTCGCTGAGCAAGACGCTGCGGTTCAAGGCGATCCCCGTCGGGAAGACCCAGGAGAACATCGACAACAAGCGGCTCCTGGTCGAGGACGAGAAGCGCGCCGAGGACTACAAGGGCGTCAAGAAGCTGCTGGACCGGTACTACCTCTCCTTCATCAACGACGTCCTGCACTCGATCAAGCTCAAGAACCTGAACAACTACATCTCGCTGTTCCGCAAGAAGACACGGACCGAGAAGGAGAACAAGGAGCTCGAGAACCTCGAGATCAACCTGCGCAAGGAGATCGCGAAGGCGTTCAAGGGCAACGAGGGGTACAAGAGCCTGTTCAAGAAAGACATCATCGAGACCATCCTGCCGGAGTTCCTGGACGACAAGGACGAGATCGCGCTGGTGAACTCGTTCAACGGGTTCACCACGGCCTTCACCGGGTTTTTCGACAACCGGGAGAACATGTTCAGCGAGGAGGCCAAGTCGACCAGCATCGCCTTCCGGTGCATCAACGAGAACCTCACCCGCTACATCAGCAACATGGACATCTTCGAGAAGGTGGACGCCATCTTCGACAAGCACGAGGTCCAGGAGATCAAGGAAAAGATCCTGAACTCGGACTACGACGTGGAAGACTTCTTTGAGGGCGAGTTCTTCAACTTCGTCCTCACCCAGGAGGGCATCGACGTCTACAACGCCATCATCGGCGGCTTCGTGACGGAGAGCGGCGAGAAGATCAAGGGCCTCAACGAGTACATCAACCTCTACAACCAGAAGACTAAGCAGAAGCTCCCGAAGTTCAAGCCGCTGTACAAGCAAGTCCTGAGCGACCGGGAGTCCCTCTCGTTCTACGGCGAGGGCTACACGAGCGACGAGGAGGTGCTGGAGGTGTTCCGCAACACGCTGAACAAGAACAGCGAGATCTTCAGCTCGATCAAGAAACTCGAGAAGCTGTTCAAGAACTTCGACGAGTACAGCAGCGCCGGCATCTTCGTCAAGAACGGGCCCGCGATCAGCACCATCAGCAAGGACATCTTCGGGGAGTGGAACGTGATCCGCGACAAGTGGAACGCCGAGTACGACGACATCCACCTCAAGAAAAAGGCGGTGGTCACGGAGAAGTACGAGGACGACCGCCGGAAGTCCTTCAAGAAAATCGGGAGCTTCAGCCTCGAGCAGCTCCAGGAGTACGCGGACGCCGACCTGAGCGTGGTGGAGAAGCTCAAGGAGATCATCATCCAGAAGGTCGACGAGATCTACAAGGTCTACGGCTCGAGCGAGAAGCTGTTCGACGCGGACTTCGTGCTGGAGAAGTCCCTCAAGAAGAACGACGCCGTGGTGGCCATCATGAAGGATCTGCTCGACAGCGTGAAGTCGTTCGAGAACTACATCAAGGCATTCTTTGGGGAGGGCAAGGAGACGAACCGGGACGAGTCCTTCTACGGGGACTTCGTGCTCGCGTACGACATCCTCCTGAAGGTCGACCACATCTACGACGCGATCCGGAACTACGTCACGCAGAAGCCCTACAGCAAGGACAAGTTCAAGCTCTACTTCCAGAACCCGCAGTTCATGGGCGGGTGGGACAAGGACAAGGAGACCGACTACCGGGCCACGATCCTGCGGTACGGGTCCAAGTACTACCTCGCCATCATGGACAAGAAGTACGCCAAGTGCCTCCAGAAGATTGACAAGGACGACGTGAACGGGAACTACGAGAAGATCAACTACAAGCTCCTCCCGGGGCCCAACAAGATGCTGCCGAAGGTGTTCTTCAGCAAGAAGTGGATGGCCTACTACAACCCCTCGGAGGACATCCAGAAGATATACAAGAACGGCACGTTCAAAAAGGGGGACATGTTCAACCTGAACGACTGCCACAAGCTGATCGACTTTTTCAAGGACAGCATCAGCCGCTACCCGAAGTGGTCGAACGCCTACGACTTCAACTTCTCGGAGACGGAGAAGTACAAGGACATTGCGGGCTTCTACCGGGAGGTGGAGGAGCAGGGCTACAAGGTCTCCTTCGAGAGCGCCTCCAAGAAAGAGGTGGACAAGCTCGTGGAGGAGGGCAAGCTGTACATGTTCCAGATCTACAACAAGGACTTCTCGGACAAGTCGCACGGCACCCCGAACCTCCACACGATGTACTTCAAGCTGCTGTTCGACGAGAACAACCACGGGCAGATCCGCCTCAGCGGCGGGGCGGAGCTGTTCATGCGCCGCGCGTCCCTCAAGAAGGAGGAGCTGGTCGTGCACCCCGCCAACTCCCCGATCGCGAACAAGAACCCCGACAACCCCAAGAAGACAACCACCCTCTCGTACGACGTCTACAAGGACAAGCGGTTCTCGGAGGACCAGTACGAGCTGCACATCCCGATCGCCATCAACAAGTGCCCCAAGAACATCTTCAAGATCAACACCGAGGTGCGGGTGCTGCTCAAGCACGACGACAACCCCTACGTCATCGGGATCGACCGCGGCGAGCGGAACCTGCTCTACATCGTGGTCGTGGACGGGAAGGGGAACATCGTGGAGCAGTACAGCCTGAACGAGATCATCAACAACTTCAACGGCATCCGCATCAAGACGGACTACCACAGCCTCCTGGACAAGAAGGAGAAGGAGCGGTTCGAGGCGCGGCAGAACTGGACCTCCATCGAGAACATCAAGGAGCTGAAGGCCGGCTACATCAGCCAGGTCGTGCACAAGATCTGCGAGCTCGTGGAGAAGTACGACGCGGTGATCGCGCTGGAGGACTTGAACAGCGGGTTCAAGAACTCCCGGGTCAAGGTCGAGAAGCAGGTCTACCAGAAGTTCGAGAAGATGCTGATCGACAAGCTCAACTACATGGTGGACAAGAAGTCCAACCCCTGCGCCACCGGCGGCGCCCTCAAGGGCTACCAGATCACCAACAAGTTCGAGTCCTTCAAGTCGATGTCTACGCAGAACGGGTTCATTTTCTACATCCCGGCGTGGCTCACCAGCAAGATCGACCCGAGCACGGGCTTCGTCAACCTCCTGAAGACCAAGTACACCAGCATCGCGGACAGCAAGAAGTTCATCTCCTCGTTCGACCGCATCATGTACGTCCCCGAGGAAGACCTGTTCGAGTTCGCCCTCGACTACAAGAACTTCTCCCGGACGGACGCCGACTACATCAAAAAGTGGAAGCTCTACAGCTACGGCAACCGGATCCGCATCTTCCGCAACCCCAAGAAGAACAATGTGTTCGACTGGGAGGAGGTGTGCCTGACGAGCGCCTACAAGGAGCTCTTCAACAAGTACGGCATCAACTACCAGCAAGGGGACATCCGCGCGCTGCTCTGCGAGCAGTCCGACAAGGCGTTCTACTCGTCGTTCATGGCCCTGATGAGCCTCATGCTCCAGATGCGCAACAGCATCACCGGCCGGACGGACGTGGACTTCCTGATCAGCCCGGTCAAGAACAGCGACGGCATTTTCTACGACAGCCGGAACTACGAGGCCCAGGAGAACGCCATCCTCCCCAAGAACGCCGACGCGAACGGCGCCTACAACATCGCGCGGAAGGTGCTGTGGGCCATCGGCCAGTTTAAAAAGGCGGAGGACGAGAAGCTGGACAAGGTCAAGATCGCCATCAGCAACAAGGAGTGGCTCGAGTACGCGCAGACGAGCGTGAAGCACTGA

>Terminator - Os.LTP

TAATCGATCCTCCGATCCCTTAATTACCATACCATTACACCATGCATCAATATCCATATATATATAAACCCTTTCGCACGTACTTATACTATGTTTTGTCATACATATATATGTGTCGAACGATCGATCTATCACTGATATGATATGATTGATCCATCAGCCTGATCTCTGTATCTTGTTATTTGTATACCGTCAAATAAAAGTTTCTTCCACTTGTGTTAATAATTAGCTACTCTCATCTCATGAACCCTATATATAACTAGTTTAATTTGCTGTCAATTGAACATGATGATCGATG

**Supplementary Material S3** **|** Genomic target sites used for editing in this study.

| **Name** | **Sequence (PAM is in red)** | **Coding/Non-Coding** | **Chromosome** |
| --- | --- | --- | --- |
| Zm.7.1c | TTTAGTATAATATGATGGCATGCCCTC | Non-coding | Chr10 |
| Zm.7.1b | TTTACTGACGTAAGGTATGGTTTAATC | Non-coding | Chr10 |
| Zm.Brm3_3170 | TTTAGAATCCACGACATGCAAGAGCTC | Coding | Chr04 |
| Zm.Bmr3_2691 | TTTCCGGCAGCGCGTCGTAGCAGTTCT | Coding | Chr04 |
| Zm.01DKD2.1G000028 (gRNA01) | TTTCGCTGGAGCTATGTGCATGCACAT | Coding | Chr01 |
| Zm.01DKD2.1G000075 (gRNA02) | TTTGGTGTGCCCTGCAAAGCAGGTAGC | Coding | Chr01 |
| Zm.01DKD2.1G000884 (gRNA03) | TTTGGAGCCACGAGATCTTTGTCGCCG | Coding | Chr01 |
| Zm.01DKD2.1G002753 (gRNA04) | TTTGGATCGAGGAGGAGATCACGGTGG | Coding | Chr01 |
| Zm.01DKD2.1G004046 (gRNA05) | TTTAGCATGTGTATGTGACAGGAAAGC | Coding | Chr01 |
| Zm.01DKD2.1G006197 (gRNA06) | TTTGGGCGATTAGAACAGCGTTTGGCC | Coding | Chr01 |
| Zm.01DKD2.1G006758 (gRNA07) | TTTGGACTGAAGGCCATCTATGACCAC | Coding | Chr01 |
| Zm.01DKD2.2G007640 (gRNA08) | TTTGGGTGGACATAGAACCGCCACGAC | Coding | Chr02 |
| Zm.01DKD2.2G007950 (gRNA09) | TTTGGAAATCCATCACCGAGACTGGGC | Coding | Chr02 |
| Zm.01DKD2.3G013783 (gRNA10) | TTTAGCAGAGCGGAAGATTCTCGTGAG | Coding | Chr03 |
| Zm.01DKD2.3G016048 (gRNA11) | TTTGGAGTGACGTCAGGTGTGTGAAGC | Coding | Chr03 |
| Zm.01DKD2.3G017216 (gRNA12) | TTTCGATCGTGCCAAGGGAGGGCTTGA | Coding | Chr03 |
| Zm.01DKD2.3G017479 (gRNA13) | TTTAGGATGAGCGGCGGTAACGACCCC | Coding | Chr03 |
| Zm.01DKD2.4G018411 (gRNA14) | TTTAGAATTAACCGCCCCTGAGGCAGT | Coding | Chr04 |
| Zm.01DKD2.4G018724 (gRNA15) | TTTCGTCGTCGCCTGTAGAGTGGAGAC | Coding | Chr04 |
| Zm.01DKD2.4G018966 (gRNA16) | TTTAGCTATATCATCGCTGATGCGCGG | Coding | Chr04 |
| Zm.01DKD2.4G022503 (gRNA17) | TTTGGAATCGAGACACAGCCGGCCACA | Coding | Chr04 |
| Zm.01DKD2.5G025731 (gRNA18) | TTTCGTAATAGGTACAAGCATCAATCA | Coding | Chr05 |
| Zm.01DKD2.5G026529 (gRNA19) | TTTGGCTGGAAGATTGGAGAGCTTCCT | Coding | Chr05 |
| Zm.01DKD2.6G028665 (gRNA20) | TTTGCTAGCAGCTACCGTAAACCAGGC | Coding | Chr06 |
| Zm.01DKD2.6G030630 (gRNA21) | TTTGGCTCGAGGAAATGTTTCATTATC | Coding | Chr06 |
| Zm.01DKD2.7G032708 (gRNA22) | TTTAGATGACTGCAGTAGCGCGAACAC | Coding | Chr07 |
| Zm.01DKD2.7G034607 (gRNA23) | TTTATATATACATCCGCCGGCCAATCC | Coding | Chr07 |
| Zm.01DKD2.8G037603 (gRNA24) | TTTAGGAGATCAAAAGTGAGGTACAAC | Coding | Chr08 |
| Zm.01DKD2.9G040323 (gRNA25) | TTTAGATCAGCTTGGGATGGCAGCGAG | Coding | Chr09 |
| Zm.01DKD2.9G042491 (gRNA26) | TTTGGGAGGGCAACACGAGGATTAGGT | Coding | Chr09 |
| Zm.01DKD2.9G043294 (gRNA27) | TTTCACCGTAGCATGACTGCATGAGAC | Coding | Chr09 |
| Zm.01DKD2.10G045914 (gRNA28) | TTTCGCTTCGAGAACCTGTTGCTCCAG | Coding | Chr10 |
| Zm.01DKD2.10G046041 (gRNA29) | TTTCAACGGAGTCAGGTGGCGTCGTGC | Coding | Chr10 |
| Zm.01DKD2.10G046376 (gRNA30) | TTTGGTCAAGCCTGCATACCACGACAC | Coding | Chr10 |


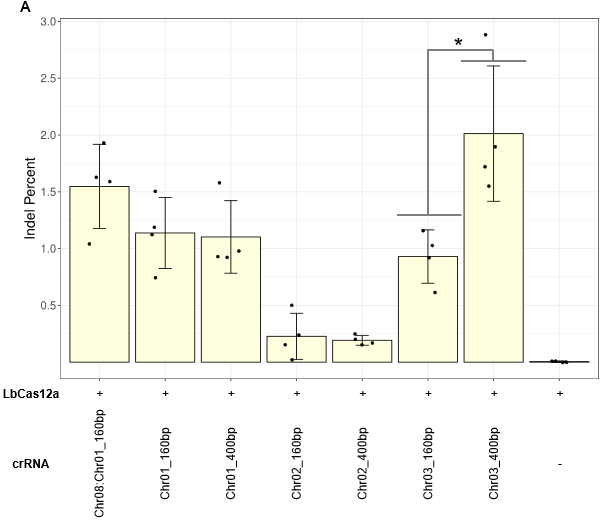


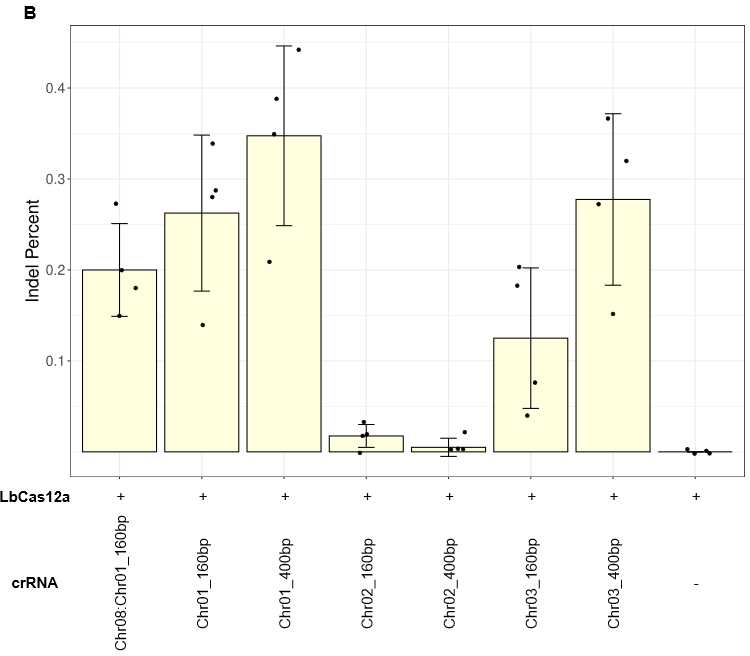


**Supplementary Material S4 |** Editing rates at the Zm.7.1b intergenic **(A)** and the Zm.Bmr3_2691 genic **(B)** CRISPR/LbCas12a target sites in maize protoplasts when the crRNAs were driven by different size variants of three endogenous maize promoters from chromosomes 1, 2, or 3. A chimeric maize U6 promoter (Chr08:Chr01) was used as a positive control. The negative control (rightmost bar) included LbCas12a but lacked any crRNA cassette. Bars are averages of four replicates (dots); error bars are standard deviations. Significant differences are marked with * (Kruskal-Wallis rank sum test followed by Wilcoxon rank sum exact test, p < 0.05).

**
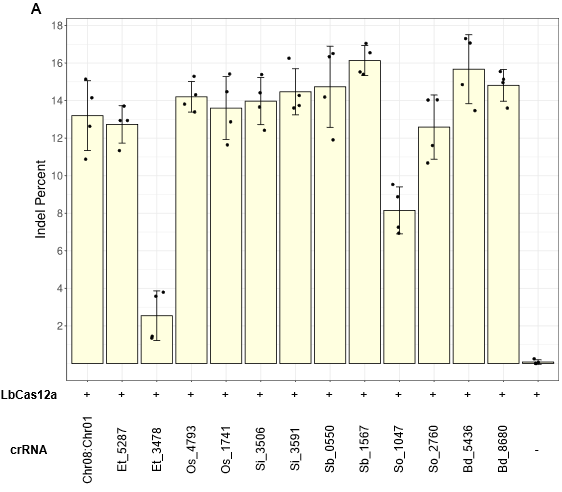
**

**
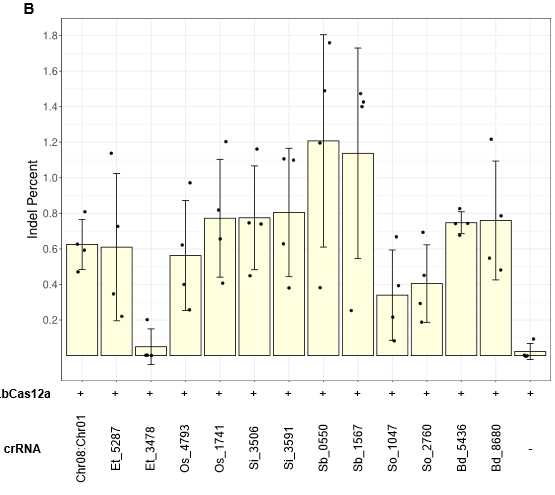
**

**Supplementary Material S5 |** Editing rates at the Zm.7.1c **(A)** and Zm.Bmr3_2691 **(B)** CRISPR/LbCas12a target sites in maize protoplasts. The crRNA cassettes for these sites were driven by twelve U6 promoters from monocot species *Eragrostis tef*, *Oryza sativa*, *Setaria italica*, *Sorghum bicolor*, *Saccharum officinarum* and *Brachypodium distachyon.* These promoters were representatives of a panel of 66 native promoters used in the training set for a machine learning model to develop computationally derived Pol III promoters. A chimeric maize U6 promoter (Chr08:Chr01) was used as a positive control. The negative control (rightmost bar) included LbCas12a but lacked any crRNA cassette. Bars are averages of four replicates (dots); error bars are standard deviations.

**Supplementary Material S6**

Computationally derived 500 bp Pol III promoters for driving crRNA transcription in maize.

>GSP2230

CTCTTCTTTTGTTTTTTCACTAGGTTGCGAATCTTTACCCACTCCCAAAAAATTCAGCTGTGCCGCGCTTGTAGTTTCAAAGTTTTGCGAAGATAACAAGCCAGTAATTCAAGAAAAAAAAAGTTAAAGCAAAGCACTGCTGAGACGATCTGGAGTACGTACTACTGCGTAACACACGTCAGAATTTCTGTTTTCTTATACACGTAACAGCTTGCAACCTTTGCAACAGTGCACCGCAGGACACAGTTACAGTGTTCGCAATAAGTACGTTCAAAAATTCTGGACCGAAAGCAAATACGAAGCAAAAGCCCACTAAACCAGCCCACGGAGAAGGAAAGCGAAGAAGCAAGCCCGTGTACTATATAAATCCGTTATACTGGCGCGACTTCGGTCGGTAGCGCAACGCGGGAACGAGCGGGGAGGGCAACGAGGTTTAGTACCACATCGATCGGCAACGCGAAAGCGACCCACCTTATAACCGAGCGCGCGCTGAGCCGTTC

>GSP2231

GAGGTTCAGCCTTGCTTTTGCTTTCTACTTGCGTTTCACACTTTCTTCAACGTAAGCTGTACAACAATCTTGCCACTGCCTTCAAGAAAAAAGGAGAGAGTTTCTTCCTCTGGTACTGTGTTTCTCGCCGAACTCAGTAATCAAAGACGGAATATTTCACAAGAGTTCCTTACGAGCAAAGTTCAGTCGTTGTGCTTTACTCACAGCTCCAAAAAAAGAGAAGAAGTGTGAGTTACTTCTGTTTTTAGCAAGTGTGCGTGGCGAAAAGGGCCTGCTGAACAAGGAGAACGAAATAAGTCGCGGGGGGAGCCCAACTGGGCTCCCACCTTGCTCAGGCCCACGAAGAAGCCCAGTGACACGCGCGCTTCAGCCCACTGCACGGCCTGGTTGGAGAAGCGCGGAGCGCGGTGCGGGGCGGGGAGAGGCTCGAGGTTTAGTACCACATCGAAAGCGCAGCTAACGACAGACCACTTTATAAGCCGAGCCACCCTGCTGCTCTC

>GSP2232

GGGAATTAGTTACGCAAGTTACGCAAACGTCTGCCCTGCTTCAGCTTAAAGGGAAGCTTCTTCGCTGTGTCTGCTTGTACTTCGTGCTCGCCGGCGTACTGAAATCTTGTAACTTTTCTTTATTAGGTTAATTCGGCGAGATTAGGTTCTACGGAGATACTACTGCGTACGAAATTTCGTTTTTTCTTAATAATTTAGGAATAGATTGGAAACACGGCCAGCACAATCAATCAGTTACCCTGGCCCAATTCCGCGTTGAGAATAGAGGTGGGCCTGGAACTCTTTTTTTTTAAAACGAAGCCCTAGTGGCACTTCGCAAGAAGGAACAGGCCGTAAGGAAGGCCCAATACGCCTCCGAAGGCCCACGCGCTGGGCGCTGCGCTGCGGAGCGCGCGCTTACGGGAACGAGCAACGCGCTAGGGGGCGTTTAGTTTAGTCCCATCTCGGCAACGCAAAGCGAAACAGACCACTTTATAAGCCCGGAAGCGAAGCCTGCTCTC

>GSP2233

CCTAAACTTGCCACGAGCGTACTTGCTTTCTCTTCTTTCTCTTTATAAGGATATATCACGGAAGGGAAAAACTCTTCGTGCTCGTTTCTTTCAAGTCAAAGTCGGTCTACGTTTTTTCTTTAGGGATTAGGGCTGTGCTTTGAAAAAGCACGTAATATCTAAAAAGGTCCGTGTCTGTGATTTAGCAGTTCAATCCAATCAGCACTTCTTTTAACAAGGAAAAATCAGAACTTCTGTGCAAGTGTTTTGTCAAGCTTAAAAGAATAGAGAGAGGCTGAGGCGAGAGAGAATTTGGTCGCAGAAAAACACAAAAAGCCCAACTGGGCTGTGGAAGCCCACGGCCGTGCGCTCAGTTCTACCGCGAAGCCCAATCGGTGCGCTTCCTGCAGCGCTCTAGCGCGCTTCGGTGGAGCGCGCGCAATCGAGCGAGAGTTTAGTACCACATCGGCCAGCAACGCGAACGCAGACCAGTTTATAAGCCGCGCTGCTGCAGCACCCTC

>GSP2234

CAACCAGCGGCGATTTTGTTGCGAGCAGTCAGAATCTTTCAGCTTATTTCTTCGATTTTCTCTTGTCAGGCTGTGGAATCTTTTGTAAGAAAAAAGTGACTGCCGGCGATAAGCCTACCACAAGGGCGCGGCGGCCCAGCAAGTTGTAATCTGCTGTAATAAATAATAACGTAATAATACTCCCGGGATACAATCTACCAGAATAAACCTTCCTTCCCTTCTTCCAAATAACGATTTCTGGTTCGCAATCGACCGAGGTGGAGAGTTACACAGCTGGGAGGAGGCAAAAGAGGCTTGGTCGAGGCGTGTGCTCTTCGTTAAGGCCGCCGCCGAACAGGCCTCCGAAGTGGAAAGCCCAGCCCACGCGCTGGCCCAAGTCAGCCCACTTATAAGCAACGACGCGCTAGGCAACGAGCGTTTAAGGCAACTCGTTTAGTACCATATCGCTAGCGAACAATCCACAAGACCAGTTTATAACGCTAGCTTAATCTGCTGCTCTC

>GSP2235

TTTCCTTTCCTCTGGAATTTCTCGTCCTCGATCACACGTACAAATACCAAAAAGGTCGAAAACAGCACTGCTTCAAGTTTTGCACGCCCAAGAAAAAGGTCTTTCTACCGTTGTCTTCCTTAGTCTGAATTTTCGTTGAATAAGCTCAACTTGATTTGTCAATTTTTCCCGGGACGTATCAGTGGCGGGTTCAATTATTTTTAGTGTGGGGATAGTACCAGCACCAGACCGGTTCGGGCAACAAAGAAAGAAAGTACCTGTTGACGGTGGCAAAAGGAGGAGAAAAACACTTCCCAATTTAGTGGCAAAAAGCCCGTGGGCCAGCCGCGGCCGTTGGGCTGGGCCCACCACGTGCGCTTATTCAGCCCAATACCGTTATTGAGCGCGCTCTGGAGAAGTGGAGCAACGAGCGCGGGGAACAAGCAGAGGAAGTTTAGTACCACCTCGAGCCAGCAAACGCGAAACGACCACCTTATAAGGCGCTGAGCTTGTGCTGCCTC

>GSP2236

CAACTTAACTTGCCAACCTAATCAAGCTAAGAAATTCTTCAACGTAGTCTCTCTCTGATAAGCTGAATTTCAGACTTCGCGAAGAAAAGCTTACCACCTCACTCTTATCCGTCGCTAAAGCTTATTAGGGAAGCCTCCCGGAATAATTTTGATTTCGTACTAGTCGTGTTGAATTTCTACGTATCGTGAACTCTTAGGAAATCAATCAGAGCTAATTTTCTACTTACACGGGCGTTTTTCGCAGTGTGTACAGAATAACAACGTACAGAATACAACAACAAAAAGCAGCACGGAAGCCCAAGCCCAAGGCCCAATTAAGGAAGGAAAGGTTTGCTGAAGCGTGGTGACACGCGGGCCGTTACCGCGCTGGCGCTGCGGCGCTGCGCTGCTTCGTCTCGTTAGCGCGCAACGAGCGCGGGAGGCAATCAGAGGTTTAGTCCCATCTCGGCTAGCGAAGCGCAAAGGAACCAGTTTATAACGCTGTACGGAAGCGGAAGATC

>GSP2237

GTCAAGCACGGCGATTCTCTGTTTAGCTACCCGCTTAAATATCTGAATAAATAAGCCTTTTGTTCAGAGGAGTACCTCAAATTCTTTCTCAGGCCCAAGTGTGATTCGGTGTCTGGTACTGTGTTTTTCTAGAATTTTTTTCCTGGACGATACCAAGTTTAGTACGGAGTAAGTCGTGTGAATTTTTAGTGCCGTGAGACTCTACAATCAATCAGACCTTCTTTTTTCCCTTTGTTCGCAGTGCACCCAGAAAGAATACGTTCGTTCAAAAAAGACTGGGCCTAGTGTGCAACGACGAGGCCTCTGAGAAGCCCACTTCCTCACAGAGAAGCGGGAAGGAAGGTGCCCACAAGGTCCCGAGCGCGGCCCACTCTGGCCTCCTGCGGAGTCAGCCCAATAGCGCGGGAACTAATAGAGCGAGCAGAGTTTAGTTTAGTACCACCTCGGCTAGCGAGAAAGACCAAGCCCACCTTATAACCGAGCACCGGAAGCGCTGCCTC

>GSP2238

TGTGTGCTGTTTAGACTCGTCTGCTGCTTTCGGAGGTGCTTCAGAAATTCTTCTTTATAATTCTCACGGAACAATCTTTTGGACAAGTCACTCGAAGAACCAAGTTTAGGAGCAATAGGAGGGCACTCAGAGTGACAAAAGAAAAAAAGAGACGGAATAATTTCGTACGGAGGCCAATTTCTGTTTTTATACAACTATCAGAGTGTGAGAGAATTTCTTTTTAGCACCGTGAGAACTTCACCAGAGGAAGATCCTGTTCGTACTTAGAGTTTAGTCACGGTCGCTCTGTTTTTTTAGTGGGGAAGCCCACTCACAGAAAGGACAGAGAGGCCCACGGCCGCTTAAGGTCCAGCCCCGCGGGAGCGCGGTGCGAGGCCCAATCTGGCGGGCTCGGTGGAGCGCTTACGCAAGTAACGCGCGCTGGAGTTTAGTTTAGTACCACATCGCCAGCAAAGGCTAGCGCAGACCACTTTATAAGGCAAGCTGGAAGCTGCTGCCTC

>GSP2239

AGTTACGCTCGAGGAATTTGTTGTTGCGAATCTTTCACTAAGAATTTCAGAATTTTCTCTTCTGCTTGGGCCGGAGCCAAAAGCTACAGAACTGCGGCGTTTCTACAAGCTTACTACGTTTACCACGCGCGGAACTCAACTTTTTTTAGTCTCTGTATATAACAGTAAATCGGACGGACGCCGTATTTCTGTGGTTTTTTTTAGTACCAGCACCAGCACACGGTTTTTTGGCTACGCACAATCGGATACGCAGGCGACCAGCCCAATTACAAGGCAAAAGAGGACGTCAACAAGCAGTGCCCACGTCGGGCGTAGAAGCCCAATTGTCCCGTGGGCCGTGGGCCGGCCCGTGCGAGCAGCCCACCGCGGGCGCACTGCAATTGCGCTTATAGCCCAGTTCGCGCGCTTGTGCGGTGCGAGAGCGAGAACAGTTTAGTACCACCTCGTCCAGTCACAAGGACGCAGACCACTTTATAAGCGCGCTGCTAGCTACTGAGATC

>GSP2240

CCGGCGATTGTTCAGCGGCGATAGGTTCTAAGCAACAGTCAGCAACGTACCAAACAAAAAAACACCTTGTGTGCCTTGTACTAGTTGTACTCAAGCTACAAGAAAAAGTGTGCAAAAATAGGGAGTACCACGGGGATTAGTAGTTCCTGAGACGGAATAATCACGTAACCAATAATTTTTCCCGGGATTTAGCAAGTTCGTGTTCGTGAAGTTTTTTTTTGGGAAAAAGACCGTGAGCGATTACAATAATATCAAGTGTTACTCTGTGTTGACGGTGGGAGGAGAAGCAACGAGGCGTAGTGGGCGTATCAGCAAAGAGCTTGCTCCGAACCAGCAAGAAGGTGCAGCAAGCCCACTGGCCCCGCGGTCGTGAGAGCGCGCTGCGCTTGCGGGATCCGATCGGTGGAGCAACAGCGTTGCGCTTATTTTAGTTTAGTCCCATCTCGTCCCAGCAAGCCAGCTTATACCACTTTATAACCCGCGCGCGCTGCCTTGTGTTC

>GSP2241

ACGTATTCAATTCAATCTTGCTGGATTTCTAAGAGGTTCTCACCTGCCAAAAAAAAATTCTGCCTTCTTTCTCACCTTGTGCTCGTTTTGTCTACAAGCAAGATACTGACGTTTAGTGTTTACCTTGTAGACCTTGTTTCAACTTTGCTGAGACGCGGAGTACTAGCAACCAAGAGTTCGCCAATCAGTTCGTGAGTTTTCTACGTACTTCTTTTTCTTCTACTTGAGCAAGTGTGTGCACCGCGTTTTTTAGCGTTCGCACGTTCACAGCCCAGCTGGGCCCAATTTGTTTAGTGTGTATCCTGAGCCAAAAAGCCCAATCAGCCCACTGGGCCGAGTGCTGGGCCTCCGCGCGGGAAGTACCGCGCTGCGAGAAAAGGGCGGTTTGGGTGTAAGGCGCTTAACGCGCGGAGGAAGGTTTAAGCGGGGGCGTTTAGTCCCATCTCGGCCAGCCAGCAACGCGCAGACCAGCTTATAAGCTTACCGAGCTGCTTTCCGTC

>GSP2242

TAGCAAAATCAAGCCGAAGGAATCTTGGTCAAAGGTTGGATAATTTCTCCCAAAAATCTTGTAGGAGTTTTCTTCACCGGAGTACCACCTCTTTCTTTCTCGTCCACCTCAGCTGTGTTCTTGTTTTTTTCTACTAGTAATTGCTGAGACGAACTGCAAGACGGAAATCGGGACGTTGTTTAGCAAAGCAGTCACTATCAGTTTTCTTATCTACGGTTTTATACAAAAAGAGGTATAATAACAGTGCAATCGCGAGATAGTACTTTAGTAATAGAGAAAGCACTCGGAGGAGCCCAACAAGGGCAAAAAGCCAAAAGCCCAGCCCAATACTGGGCCTGGGCCCACAGACGCGCGGGCGCGGGCCGTGCGCTGCGGCCCCGAGGCTGGTTCCTGCGGAGCGTTGCTGGGAGCAAGCGGGGCAACAGGAATCGTTTAGTACCACATCGGCAACGCAAACGACCAGCGACCAGTTTATAACGCTGCCAGAGCGGAGCGCTGTC

>GSP2243

CGTCTCTGTGTGCACCGGAATCTTGCTAGAGAGCTAATATCTGCTTCTTTTTCTCTGGTCTGCTTGGTTTTTGTACCTCGACCTTATAAGAACTGAAGCTTACTTCCAGGCCTATTAGGGAAGCTGAACAAAGAATCACTCCTCAGCAAGACGGAAAGATTTTGAGGAGAGACGTGAGAGTTCGCTCGAATAGGAAACAGAGGTTAATTGAAAAAGAAGTGTGTGACTTACAACGATTACACAGTACAGAATACAGTGTCAGGCCCCAACGGTGGGAGAAAAGGCGTAGGCCTGGGTTAGTGGACTGCTGGGCCTCTCTGGGTACGGAGAAAGCCGAAGAAACAAGAAGAAGGCCGTGTACCGCGCGGCGCTGGCGCAGAAGGCCCAATTCGCGCTCTAGCGCGGGGCAACGAGCGGGGCAACGCGCTAAGTTTAGTACCACATCGGAAACCGAACAGCTAACGACCCACCTTATAAGGTGGTAACTGCAGCTGCCACTC

>GSP2244

TTGTTAACAACCAGCGGAATTTAATTCTGCTTCAAGTGCTTTCTCCCAAAAGAGCTAGAGTAGAGAGGAAATCAAACTCTTTCTCGAAGACTTCTGTACCACGCGCGGCTGACGGTGTCAAAACAAGGGCTGAAAGAAAAAGCTCTGTGGCTCAGTCGTAATAATTGTTAGCGCCAATAAGCCACACACGTTGTGTTCGTTCGTACCAGGCGCCAAAGAGAAGTGAAGGTTCTTGAGCACCGTGAAGATTTTTTTGTCAAGCGAACTCTTCAAAAAGAGCAAAAAGGCCTACGAGCCCACTTCCTCCGCTCTGATCGAAGCCCACTTCGAGAGGCCGGGCCGTAAGCAGCCCAATACGAGCCCACGCGGGCTGCGGGGGGCGCGAAAAGGCGCTGCGCTTGGGAGGCAACGAGCGCGGGGGCAACAGTTTGTTTAGTACCACCTCGGCAACCCAACGCGAGAAAGACCACCTTATAACGCGCGCGCTTGAGCTTGTAGTC

>GSP2245

GATATTTTGCTAGCTTTTGTTAATTCTATTAATCGTTGGATTCGAAATTCTTTTACAAATCAGTTACGAGCTAGTTTCTTCGCCCAGCACAAAGTGTTTAGTCGACGGAATTTCGGTGACAAACTTTACCACTGGGCTTCCTGAAAAAGCAGTTAGCTTACTATCGTTGTTCTGTCAATTTTAGCAAACAAATTCTTAAACGTGTTTTTTATACAACTCGGAGAAGTGACCTGCAGTTCGCACAGTTACTTTTAGTAGGAGATAGTACACAAAAAATTCTGGCCCACGGAAAGCAGCAAGGCGTGAGGGTTGGTCGTGGGCCACGGAGGTTGGGCCGTTCTAAGCAAAAGATTCGGAAGTGCGCGGGAATCGGTGCGGCGGCCCAATCACGGCTGGGGAGCGCGGGAACGAGCAACAGTGCTCAGAGCAGGTTTAGTCCCATCTCGTCCAGTCACAAAAGGAACGACCACCTTATAAGGCTCGGCTGCCACAGCCGTTTC

>GSP2246

CTCGAGGCAACAACCAACCTCGCGGCTAGGTGTTCAGCTTTCTCTGAACAGTCAGCTGTCTACGTACACCAGTTTCTTCGTGTCTTGTCTTGTGATAAGCCTACCACCTTGTCTGTTCTTTAATTCTCGCCTTCTGTAATAAGCTCAAAGCTAGAGCTAGCAGTCTGTTTAGTTCGCCAGAGAATTGTGGTTCCAATTGAGAGGTTCCAAAAGAAGTGAAGTGAGCAACAGTAGGACACAATTCCAGCCCAGGACCGAACTCTTCCTGTACAAGGCCGGGAGGAGCTGGGCGTAGTGGCTCTGGACTGGGCTCTCTACTGCTGATACGGCCGTAAGGAAGGTTGCTAAAGTTGGGCGCGCACCGCGCTGGCGCTGGTCGTGTCAGCCCAATTAGCGCGCTTCGGTGGAGAACTGGTTGCTTATCGAAGAAAGTTTAGTACCATATCGGCCAGCTAGTTTAGAGCGTACCAGTTTATAACCCCGCGGAGCCGCTTAATCTC

>GSP2247

TGTGTGCCCTGGAATTTCAAGCAATCTTGCGTTAACAGTCAAGAAAGAGAGCTGTGCCCTGTGCCCTGCTTGTAACAATCAAAGTTTATAAGCAAGCCAGCTGACAAGCTGTTTTTTTTTACCGTTGCACTGGGCGGGCACTCAGTTCGTGTGCAGTGATTTTGAGGAATAATTGGGCGGGACCAGGAAACGTAACAAAGCAGAGTTTTTATACCTGTGTGTAAAATACAACTTCTGTGTACTTGAGATAGCACCGTCCCCGGCCCAGCAAAAATTCTGGCAAAAAAAGAGGCTTCCCACTAATTGAGAAGAAAAGAAGAGGCCTAAGAAACGCCCGTGGGCCCACAAGGCGGGCCGCTCGAGCGCGGCCCACTTTCGAGCAGTTCGTCGAACGAGAGCGCGGGGAGCGCGGGAACAGTGGAGAGGACTCAGTTTAGTCCCATCTCGAGCAAGGAAACAGACCAAGACCACTTTATAACGTAGCCTGCTCTCCACCCTTC

>GSP2248

GTGCACCGTCGTCGGAATCCAAGCAGCGGCCTTGCTTTAGACTAACCAAAGGTCAGTTTTCAGACTTCGTGCCCTGCCGGCGAGCCAGTACCACAAGCAAGATATACTCTTTGTTACCACGTACGTGTTTCACTAGTAGTAGTAAAAGCAGTAGCTTATCAGTCGTACGGAGAGACGTTTTTCCAGGAAGAGTTTGTGCCAATTCTTATTTCTTTTAGTACCTTCTTCTGGGGGCGGCGTTCGCAGAAAGAATACGTTCGCACGTAGCGAAAAGGGCCGAAAAAGGAAAGCCTGGACCAAGGAAAAACACAAAAAGCGTATCCTGGAAAGGCCGCTTCGGTTTAGCCCGTGTGCGGGGGAGCAGCCCAATACACTTTAGCCCAAATTAGCGCGGGTCGGTGGAACGCGGGGCAACAGGCGGAAGTTTAGTCGTTTAGTACCATATCGGAAATTAAGAGCGAACAGACCCACTTTATAACGCTGTAGCTACCACCCTGGTC

>GSP2249

TAGCCGTCTCTGTTTTTGATATTGTTGCGAATCGAAGCTACCACCACCTTTCTCACCGCTTGTACACCTCTTGGGCTGTGCTCGAAATCTTGTTGGTTTTCTTTCTTCACCACGTGTTCTTCGTTGACAAACTACTGGAAAAAAGGTCGTGCTGTAATATAATAATAACAAATTTCTGGAGGATTTATACACGGGAAACGTAGTACGGCTGAATCGTGTCTCCAGCAGACCGGGCACAGTGTGTCAAGCGTTGACGTCAAGCGTTCGCCGCAGGCCTGGCCCAATCTGGGTCAACAGGGCAAAGCCCTCGCAAGAAAAGAAAGAAAGAAGCCAGCCCAATCGGCCCAGCCCACGCCTCAACAACGAGCCCACTGCGGCCTTGCGGGAGCGTCCTTCGGTCGGTGGAGCGCGTTGCGGGGTTTAGTACCTCAGTTTAGTACCATATCGGCTTAGCAAGGAACAAGGAACCAGTTTATAAGCTGCAGCTGTGCTGCCTGGTC

>GSP2250

TAAGCAGCCTAAACTTGCTACTGCTGCCAAAAATATCTGATTACACACTTGTAGGTTATTTCGATTTTTCTTTCTTCGCCCAAGTTGTAAAAAGCGATAAGGAGGAGCTGATTTAGTCTAAAGAAAAAGAAAGATCTGCCAGTTCACGTAGACTTGATTTTGAGGACGTGTGATTACTAAGCTACGCTCGCTAAGCTAAACGTGTGACCGGCGCACTTAAAACACACGGGCGTGTGTCACCAGAGGTATATCAAGCGCGCAATACGCACGTAGGTCCGCTGTATTTGTTAAAAAATCAGCTACTTGTTGTTGTTTGCTCCGTTGGGCCGCAGCGAAGAAGATTTGGCGGGCTTCAGCCCAGCCCGCAGCTTGCGGGCGCTTCCGCGAGCGGCCCACTTCGGTGGAGCGCAACGCGCGGGGTTTAAGCGTTGTTTAGTCCCATCTCGGAGGCAAAGCTAGCGAAAGACCACCTTATAACCGAGCGGAAGCGCTGCTTGATC

>GSP2251

AAGCAGCGGCGATTTAACTTGCTAGACTAGGTTCCAGAAGCCTAGATCAGAAGCAACCTCTTTAGTTTTTGTCCTCGACACTGTCTTGTTCTCGAAGACGGTGTTGGTTATTAGTCGTAATAAGAAAGAAATCAAAGAATTTTTTCGTAATATACTAGTCGTACGGAATTTTGTCCAGGATTTAGTTCGTTTTTCCAGCACCAGCACCAGCAGCACCAGAGTTCGTGAAACCTTCTTCTGTTCGTCACCAGAACTCTTCTTCTTCTGTGTGTGTGCAAAATCTGGGTTACAACAAGGGCGTGTGGCGTATCCTCACAGGAAAGAAAGCGAACCTTGGGCCCCGAGCGGGCCGGAAGTACCGCCACAAAGCCCACGAGGCCCACTTCGGTTCGTCTCGTCGCGCTTACGCGCGCGGGGTTTAACAACAGAGGTTTAGTACCATATCGGAAACACAAAGCGAAGCGTACCACCTTATAAGGAAAGCGAAGCGAAGCGCGCTC

>GSP2252

AGATACGGCTAGCAAGCAAGCAAAACCAACCTTGCGTTGTTAAATAAGCTGGAAATACGCGTACACCACCTTGTGCTCTTTTTCTTCGAAGAACCACAAGCTTACTCTTTCTTCGTCCCGGAACAAGCTGTGTTCCTGAATATCGACTTTTTGCTGTGAAACTCGCTAATCAGCAGTATTTCAATTTTTCTGAGAATAATCCACAGCTAATTTCTTATACAAAAAGAGCCAGGAGCAACGAAGCCTGTAGCACCGCGTTGAGAAGCTTCAAAAAGAGGCAAAAGGCGTACAGCAACTGGGATTTTGAGGTGGGAAAGCCACTTTTGCTGGGCCGTAAAGCAACAGCCCAAGGCCGTTCTAAGGCCCACTCGCTGCGCTGGCGAAGGCAACAGTTCGGTAGCGCAACGACGGAGAGTGTGAGCGAGCCGAAGTTTAGTACCACCTCGGCTGCTTAGTAGACGACAGACCAGTTTATAAGGCTGCTCGGCTGCAGCTGCCTC

>GSP2253

GGCTAGACGGAATTAATTCTCTTCTTCAGGCTGGTCAAGAATTTCTCTTTTTCTCTGAATCGCCAAACAATCGACACTGTCGATAAGAGCCACTGTTCTCGAAGAATCTTCAGCTGAAATAAGAAATCTTCTGTAATCTAGCTGTGCTGACTTAGTAATAATCAGTACGGTGTCCAGGATTTCAGCAAGTTCGTTGAGCAATTTTTAACCGGTTCTTTAACAGAGTGAAGAAGTGTCAGAAAGCTCAGTAGGAGCCGCGAGAATAGTCAGAGGAGAACCGAGAAGCCTAGTGTTAAAGGCTTGCTGGGCTGTGCAAAGAAGAAAGCCCACTTGGGCCTGGGCCTCAACAAGCCCAATACGAAAGGCTGGGCGCGCTGCGGGGGGCTTGCGGGCTTCGTCGGCGCGCTTGGGGGCAACGAGCAGAGGTTTAGTTTAGTACCATATCGCCTCGAAAGGCAAGGAACGACCACCTTATAACGCTGCAGCTGTGCAGCTTAATC

>GSP2254

GAAATCCCAAGAATTTTGTACTGTGTGCGTTGGCGATTTTCTCTTCTGGAAAAAAATTCTTTACCACCTTGCACGATAAGCCAGTATTTTGTACTAGTACCACCACCACAAGAAAAATTACTACGTGTTTCACTCAGTCGAAGATCAGTTACCAAGTTTCTTGTTCGTACGTATTAGCGTGTGCACCTGCCCCGGGAATCGTTTTTTTCTTCTTTTTTTCTTTTTTCTACCCTTCTACGCCAGGAGATAAGTGTGTTCGCACGCTTCACCTGTACAAAAAAGCAACTGGGCTCCAGCAACGAGAGCCACAATCAACTTCGTTAAGAAGCAAGAAAGATCTCCGCTAGCCCGTGTACTCGCTTCCGTCGGGAGGCCCACTCTGGCGCGCTGCGGGGAGCGGCTTGCAACGCGGGGCGGGGGCGCTAGAACAGGTTTAGTACCATATCGAGCAGTTAACGCAGCAACGACCACTTTATAACCCGCCAAACCACCCTGCTCTC

>GSP2255

TCTTGCTAGCTCTGTACTGCAGCGATAGGTTCCCAGATTTCTCTCTGAACACCAAAAAAGAAACTCAAACACCTCGAGCCAAAAGGTGAGCAGTTTCCAGTTAGCTGAAAGGCGTACTACGTTTAAAGCTTATCCTAGTAAGCACTCGCCGTAATTAGTAAATAATAATACTGTTTAGACTTAGCAGACTGTTTTTCTTATTTATATACTGTTCGCTGTCGCTTACAGAGCCAGAAGTGTGTCAGCGCCGCGAGGTGCTGGTGTACAAGAAGAGCTTTGTTAAAAAAAGGCGTATCAGCCCACTGGGCCCACCTTGGGCTAAAGCTTATAAGCGGCCCAAAGTTGGGCCTCCGCGGGCTCTACCGAAGCCCACACGGGCGGGAGCGGCCCAGTTCGCGCTGGGATTGCGCTGAGCAGAGCGTTAAGCGTTGTTTAGTACCATATCGGCTGCTCACGCGAAAGCGACCCACCTTATAAGGCGCTTAACCGGAAGCCTGGTC

>GSP2256

GGAATTTCAACGGCCCAATTGTTTTTCTCTGCTTTCGTGAATCCTCTGAACAGAAAAAAAATCTTGGGACGAGCCCAGACTAAAAGCTACAAGTGAGCAGTACCACAACGGTGTCCAAAGGCGCAGAATTTCCTGAATTGCTGCTTTTTTTCTTGATTGCTGCCACAAGTCGTACGAGAATAGTTAGCAAAAAGTACCAGCAAAGGCACACCCTCGTGAAACAAAGCAACAAAGAGGCAACAGTTTTTAGCTGGATTACAGCAAGAAGCGCGCTTCAGCTGTATCTGGCGGCCTGGACTGAGCAATCAGCTGCTGATTTGCAAAAGCGGCCGTGCACAAGAAACAGGCCGTGGGCTGCTACAACTGGGCTGGCCCACCGCGGGCTGGCCAGTTCGGTAGCGCTTACGAGCAAGCGGGGGCAACAGAGTTTGTTTAGTACCACATCGGTTGCTCAGCGCAAACGCGACCAGCTTATAACGCTTTGGCACCGTTGCGGGATC

>GSP2257

CTGGTGGAATTTTAGCCCAACCTTCTCTGCGTTAAAGGTCAAAGGTTCCAGAAGGTAGGAAGGGAAATATAAGCTCTGTACTAGTACCTCTAAAAGCTCGGCGTAGGAGGAGATTACTAGTACCACGCTTTGGTTCTTTCGAGCTTTGAAAACCAAGTTTACTATCGTACTAGCAAGAGACGGTGTCAAAAAGCACCTAAGAGCACTGCACACAAAGGCCGGTTTTTGCAAGCAGCACCAGCAAGTGTGCTATTCAAGTGTCAAAGGCCCAGCACAAGGCCCAAGCTTATTGTGTGTAGTGAGGTTATCGACTGGGCCAGCTGTGGAAAGGTGGAAAGCCCACAAAGCCCAGCCCGTCTTGGCTGGGCTGGGCGGCCCAACAGATAGCTCGGATAGTGGAGCGCAGTAACGAGCGAGCAGGACGCGCTAAGTTTAGTACCACCTCGGTTGCCAACGCAAACGCAGACCAGCTTATAAGGCTGAGCCTGCTGAGCTTTGTC

>GSP2258

TGATTACGTATCTGTGTCAGAACAGAGGTTCTTCAGTTCAGAAAGGTCTTGTCAGCCCAAACTTCCTTGCACGCCGGTGGAGACGTGGAGGCAAGACAAGTCGACGGTGTAGGAGCAATTTTTTCTTGTAAAACTTAGCTTAGCACAAGAGACGTAGTGCTGAATCTAAGCTGAACTAGCAGATACTGAATCAATTTTTCTTGCGCTTCGCTGCCAAAATTGCTGTCAACGGTTCGGAGTGCAGATAGTCCTGTTTTTTTGTAGCCGCGTTCGGAGAACTGCAAAAAAATCAGCTGACGAGGCTCTGATCGAGGAAGAAAGGCCTTGGGCCGTTTCTTCGGTTTAGAAGCCCACGCGGGCTGTGGGCGCTGGGCTGCGGCGCTGCGCTGCAGTAGTACCCAGTAACGCAGTAACGCGCAAACGTTTAGTAGTTTAGTACCATCTCGGCTGCCGAAAGCTAACGAAGCCACTTTATAAGCCTCGCGCGGAAGCACCAGTTC

>GSP2259

CCTAGCTAGCTCTGCTGCTTTCTGCTTCACACTTGCGAGGAAATTCCTAGAGTTATTTCTCCAGCTTGTGAGGCAATCAAAGTTGTAGTAAGGAAATCTGCTCGCAGTAAGGAACAGGGCACTGATTTCTCAAAGCTTTGTGTTTAGTAGTAATATACTAGCTGAGTGCTTAGCAGACGATTGGGGCGGGTTAGCAGCAAAAAGGAGCAAAGGAATCGTTTTCAGAGTTTACAAATTGAGCGAATCAGCCCAAGAATACGTGTCAAAGACTTGAGCTTATCTGGTATTATAAACCTGGTAAAGGGGGGAGAAAGCCACTTGGGCCCAGCCTAGAAAGCAAGGTGAGGAAAGGCGCGGCTGGAGAGTGCTGGCCCAATACAGTTCGTTCCGGGCTGGGGAGCGCAAGCGCTTACGCGGGGCAACGAGCAGAGTTTAGTACCACCTCGGTTGCTCAACGCAAAGGAACCCACCTTATAACCACTTATAGGCGCTAGCTGCTC

>GSP2260

CCGAAGGAATTTAAAAACTTCAAGTTCTTCCTTAAGCAGTTCTGTAAAGGAGGTTGTTTCGTAACAATTACCCAGCTTCACCCAAGGCGATAAGCAGAACTTTAGTGTAACTTTATTGCACTTGTGAGCCGAATAAGAGCTTTCGAAAAAGCTGAGAGTGCTCGTGTGAAAATAAGCTTAGCTCAAGTTTGAACACGGCCGGTAAGTTTTTCTTTTTTCTTTTTGCCGTACAGTGTGTGTCAAGACCTGTAGCAACAAAATTGAGCTTAAAAGAGTTTACTGGGCTCTGAACAGCTCTGAGAAAGCCCACGGTGGGCTGAGGCCTCCTCAACTGGGCCTAGAAGGCCCAGAAGGCCTGGGCTGGGCTTCCAGTACGCGGGTTTGGGCCAGGCGGGAGGCAACGCAACGCAGCCAGTGCGCTAAAACGTTTAGTTTAGTACCACCTCGGCTGCGTAGAAAGCTAACGACCAGCTTATAACGTAGCCGAGCCTCACAGCGTC

>GSP2261

GCTCTGTTACGCAATTCTTCTTTGCCCTGAACAGTCAGAAAATCTTTTCGTAAAGGGAATCTCTACACTCACCTTGTGAAGGTGAGTTTCCGCCCGCTGAGTGTGTGTTGACAAGCTGAACGAGCTTTTTCTCAGAAAGCACTTTTTTGAAGGGAAAGCCGTTCACAACGAAGCAGATTATAAGCTTGGCAACTGCAGACTGCACTTATTTAGTACCAGCAACCACCAGAGGCGGCAACAGAAAACGTTCTTCAATTGTTGTGGGCTGTGTTAGAGGAGCTGTGTGCTCTGAGGCTCTGGGCTCTAACAAAAGCTAAAGAAGAGCTGGGCCGGCCCAAAGAAACAGGCCGTACACGCGCTCGGCCCACGCGCTTGCTACTGGAGCAGTAAGGCCTGGGCTGGCGCTTGCCGGTGGAGAGCAGAGGTTAACAGTTTAGTACCATCTCGGCTGCTCAAGCTAACGCAGACCACTTTATAAGCTGGAGCTGCCTTGCAGCGTC

>GSP2262

GTTATTGTTATATAGCACGTTGGAGGCAGAATCTTCAACGTCTTGAGTGCAGGTCTTCTGAATAAATCTTTTTTCTCGACCAAGCCGTTGTGCAAAGGTCAAAAAAGGTGTCTAGCGGCTGTTTAAAGAAAAGGAATCAAAGGAGGAACGATTGCTGCTGCTTGCTTTGCTTTCTATCGCCAGGATTGACAGTTCGCTGAACTGTTGGCCAAGATTACAGAATCTGTGAAGAGTTTATTCACGATTAATCTAGGAGGAAGCGCGCTTTGTTGGCTGTAGGCCCAAAGCAGCAATCAACAGAGTGTGTGAGGGCCAGCTGCTGGAAGGTTCGGTTTTTTCAACAGGCCCACACGCGCGGGCTGGGCTGGGCTGGGCTGGCCACGTTCCTTCGGTAGCAGTTCGGTGGAGCAACGAGGACGGTGGAATCGAAGTTTAGTACCACCTCGGCTGAAGATACGGAGAAAGACCAGTTTATAACCTGCCTCTGGAGCCTGCTGCTC

>GSP2268

CTCGAGGAGGTCGCAGCGGCGATTTGTTGCCAACGTAACCTTCAGCTCGCGAGGCAAAAATTCTTTTTCTTCGCCGCTCAACACGGAATCGAAGACCACCTCGCTGTTTCTCGATTTTACCTTGTTTCACTCAGAGTGAGCTGAGTGAGAAAACCAATAATAACGGTGAATCAGCAGCAAGTTCAAATTGTATTGCGTTTTCTTATAAGGAAACAGCACTACTTACTTTTTAGTACCCTCGTTCAAGAAAGAAGGCCCAAACGCAAGACTGGGTCGCTGAGCAACAGAGGACGCGGTGGGCGTGTGTGCAAGGCCTCTGCTGAAGAAGACAGGAAGGTTGGGCTACTACTGCTAAAAGATAAAGCCCACGGCCCAGAAGCCCAAGATCGGTTCGGTGCTGGGAGCGCGCGCGCTTAGCGAGCAGCCAGTAGGTTTAGTACCACATCGGCTGCTCACAAAGCGAAAGACCAGTTTATAAGGTGACCTGCTCACTCACTCTC

>GSP2269

TTGTGGAATCGAGGAGCTGCTCAAAGTTCAACGTAACCTTTCAGAAGCTGAATTACAAAAGACCACCTCGACTTCTCACGGAGTACCAGCTGTGAGCTTACTCTTTAGACTTTAGTGGTGTCCAATCGACTTTTTTAAAGAAGATCTGGGTGTGCAGTTGTTCTAGACCAGCCGTACACTGAGCAAGAGTTTTTCTTTTAGTAACAGAGTGCAAGGTTAGTACAGTCAACGGCAACAGTTTAAGTGAGCTTAAAGTCAAGCGCTGGTGAGGCCCAACAAGCGTCAACAGCAACGAGAGGCTCTCTGCTGGGCCAGCAAAAACTGGAAAGCGGGCCGAAGAAACGCGGCTGGCCCACCTCTTCGCTGGCTTTTTATTGAAGCCCAGCTCTGGGTGGGAGCGCAAGCGCTTACGAGCGTTGGCCACGAGCAGAGTTTAGTACCACATCGGCTGCGTAGAGCCAGCTAGACCACTTTATAACGCAAGCTGCAGCTTGATCCTC

>GSP2272

TATAGACGATATCACGCTGCTGCCGTCAACAGAAGGTCGGTGAAGCTGAAAATTTCGAACGGTGTGTGCTAGTAGTTTTCTTTTTTAGCAAGACCACCTCTTGTTAGCTTCTTTTCAAAAAGGAACTGAGCTGACTTGATACTAGTACGGAATAACGGAATAAGCTAATTTCAGACGGTGTCTGCAAACGTTTTTTTAGTACCAGCAGTGTCGCTGAAGTGAAGTGTGTGTCCTTCTTCTACTTGCACCCAGCTGAGGCCCAAAGTGTTCGCGTACACAAGGCGAGAGGCAAACGAGGCTGGGTGGGCCCACAAAGAAAGCCACTTGCTGCTTGTCAAGCTAAAGCCCGTGCTGGCTGGCTGGCTGGCCCACAAGATATACAGAAGCGCTCTGGAGCGCGGCGCTTCTGGAACGAGCGCTAGGCAATCGAAGTTTAGTACCACATCGGCTGCTCAAGCTAGCGAACACCAGCTTATAAGCTTAACTCCGAGCCTCTGCTC

>GSP2273

TCTATTTCAAGCTGAACTGCTGCCCTGCTCGGTGTTCAGCAGAAGCTCGGCCGTAAAAGGGAATCTTGGTTGTGCTAAAAGTACTTCTGTACTAGTTACTCGCAGCTGACGCGCGCACTGAACGTACTTGTGTTTAGTAATTTCGAAAAATTCAGATCAAAAGCCGTATTCTACGTAACGATTGGGGCAGACTGCAGTACCAGCACTGACGACAGCACTTACCAGCTCTGTGAAACTTCACGAAACGCAATCGGAAAACGAGGTGGGCTGTGTTACACAGCTGGGCCTGTGTCAACAGCAACGAAGCCCACCCAACAGAGGATTGGGCCTCGATCAAGCGGCCTCACAACGTGCGGACACGCGGTGCGAGGCCACTCTGTCAGCAGAAGCCCGGCTGGGAGCAACGAGCAACTTGTGCGCTGCGCTGAAGGTTTAGTACCACATCGGTTGCGTAACGACCAGCTTACCACCTTATAAGCTTAAACCGGAGCGCTGCTTTC

**
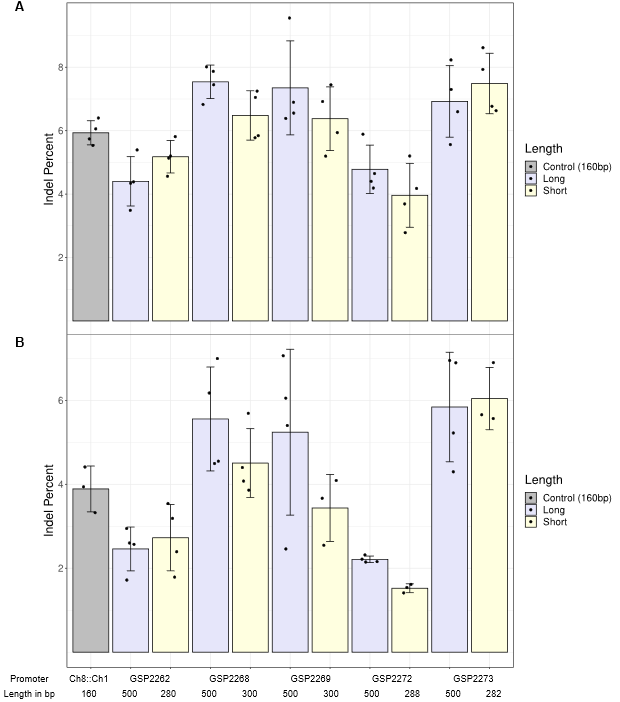
**

**Supplementary Material S7 |** Editing rates at the genic target sites, Zm.7.1c **(A)** and Zm.Bmr3_3170 **(B)** in maize protoplasts, when the crRNA cassettes were driven by two length variants of five novel Pol III promoters: GSP2262, GSP2268, GSP2269, GSP2272 and GSP2273. Bars are averages of four replicates (dots); error bars are standard deviations.

|  |  | Target: Zm.7.1c | | Target: Zm.Brm3_2691 | |
| --- | --- | --- | --- | --- | --- |
| Promoter | Training Set | mean | std dev | mean | std dev |
| GSP2230 | U6 | 6.769 | 0.916 | 0.066 | 0.032 |
| GSP2231 | U6 | 8.525 | 0.437 | 0.177 | 0.048 |
| GSP2232 | U6 | 7.744 | 1.198 | 0.276 | 0.074 |
| GSP2233 | U6 | 9.800 | 0.881 | 0.228 | 0.065 |
| GSP2234 | U6 | 7.868 | 0.519 | 0.187 | 0.055 |
| GSP2235 | U6 | 9.120 | 0.506 | 0.232 | 0.069 |
| GSP2236 | U6 | 5.429 | 0.377 | 0.067 | 0.015 |
| GSP2237 | U6 | 7.981 | 0.738 | 0.170 | 0.072 |
| GSP2238 | U6 | 7.885 | 0.688 | 0.195 | 0.033 |
| GSP2239 | U6 | 8.885 | 0.736 | 0.325 | 0.091 |
| GSP2240 | U6 | 2.108 | 0.272 | 0.006 | 0.011 |
| GSP2241 | U6 | 7.903 | 0.503 | 0.076 | 0.038 |
| GSP2242 | U6 | 8.249 | 0.949 | 0.241 | 0.080 |
| GSP2243 | U6 | 8.708 | 0.488 | 0.243 | 0.066 |
| GSP2244 | U6 | 9.082 | 0.605 | 0.300 | 0.090 |
| GSP2245 | U6 | 7.860 | 1.180 | 0.409 | 0.103 |
| GSP2246 | U6 | 4.252 | 0.701 | 0.052 | 0.014 |
| GSP2247 | U6 | 5.371 | 0.461 | 0.072 | 0.040 |
| GSP2248 | U6 | 7.056 | 1.166 | 0.091 | 0.044 |
| GSP2249 | U6 | 6.380 | 0.706 | 0.035 | 0.019 |
| GSP2250 | U6 | 6.252 | 0.725 | 0.105 | 0.061 |
| GSP2251 | U6 | 7.058 | 0.949 | 0.043 | 0.023 |
| GSP2252 | U6 | 9.846 | 0.928 | 0.171 | 0.021 |
| GSP2253 | U6 | 8.125 | 1.029 | 0.047 | 0.030 |
| GSP2254 | U6 | 4.503 | 0.733 | 0.046 | 0.008 |
| GSP2255 | U6 and U3 | 7.633 | 0.796 | 0.107 | 0.041 |
| GSP2256 | U6 and U3 | 7.549 | 1.396 | 0.243 | 0.075 |
| GSP2257 | U6 and U3 | 9.472 | 1.203 | 0.219 | 0.098 |
| GSP2258 | U6 and U3 | 8.558 | 0.668 | 0.120 | 0.059 |
| GSP2259 | U6 and U3 | 9.777 | 0.955 | 0.067 | 0.037 |
| GSP2260 | U6 and U3 | 8.555 | 1.191 | 0.163 | 0.051 |
| GSP2261 | U6 and U3 | 9.160 | 1.375 | 0.070 | 0.023 |
| GSP2262 | U6 and U3 | 6.132 | 1.193 | 0.050 | 0.044 |
| GSP2268 | U6 and U3 | 7.693 | 1.070 | 0.173 | 0.091 |
| GSP2269 | U6 and U3 | 7.720 | 1.527 | 0.153 | 0.061 |
| GSP2272 | U6 and U3 | 5.413 | 1.159 | 0.061 | 0.041 |
| GSP2273 | U6 and U3 | 7.203 | 1.245 | 0.223 | 0.114 |

|  | Zm.7.1c | | | Brm3_2691 | | |
| --- | --- | --- | --- | --- | --- | --- |
| Training Set | Median | Mean | Std. Dev | Median | Mean | Std. Dev |
| U6 | 7.692 | 7.311 | 1.947 | 0.144 | 0.154 | 0.116 |
| U6 and U3 | 8.241 | 7.929 | 1.629 | 0.120 | 0.137 | 0.088 |

**Supplementary Material S8 |** Use of U6 promoters only or both U6 and U3 promoters for training sets resulted in active computationally derived promoters. There was no clear trend favoring promoters from one training set versus the other. Upper panel: training data used for each promoter and Indel percents at two target sites. Lower panel: summary statistics for binned data from promoters resulting from each training set.

**
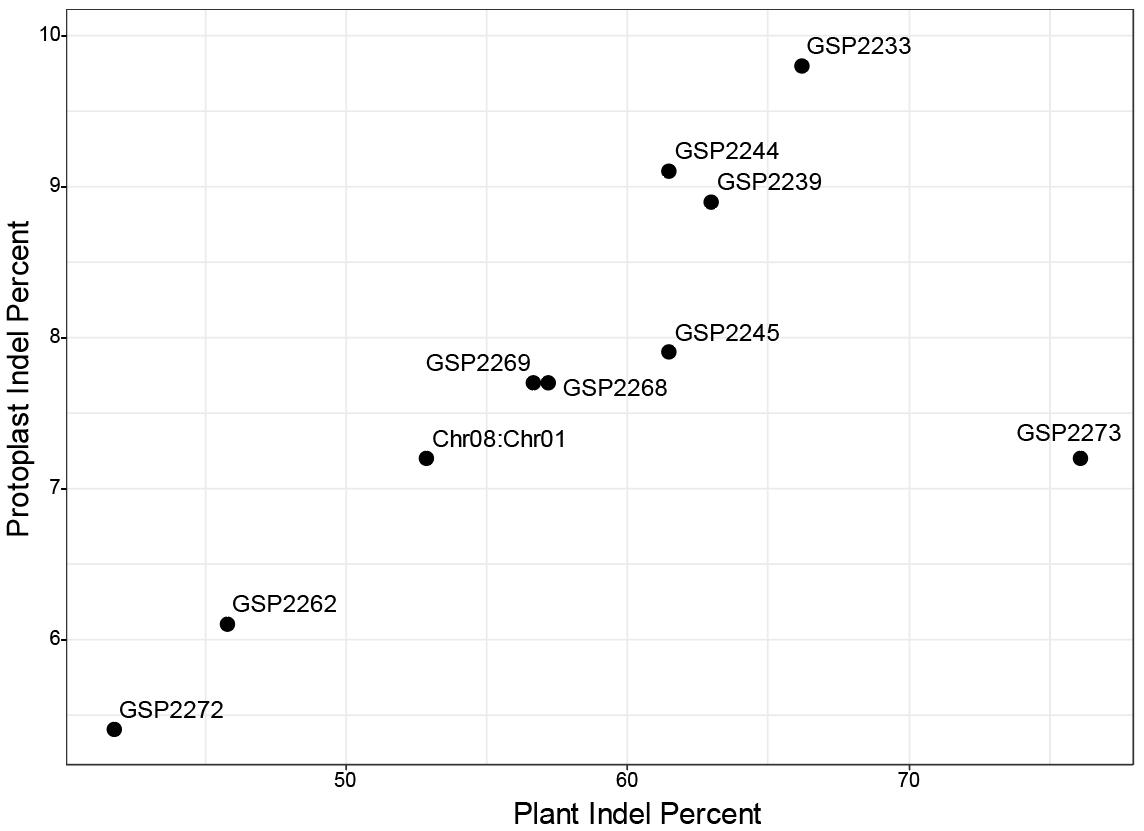
**

**Supplementary Material S9 |** Correlation of editing rates in protoplasts and in plants at the Zm.7.1c intergenic target site of maize, when using ten different Pol III promoters for driving crRNA (Spearman correlation = 0.68, p < 0.05).
